# 3-fs-resolved UV/XUV photoelectron spectroscopy in the gas phase

**DOI:** 10.1038/s41467-026-76602-4

**Published:** 2026-08-19

**Authors:** Marta Pini, Stefano Severino, Lorenzo Mai, Marta Ortolan, Daniele Mocci, Lorenzo Colaizzi, Nikoleta Kotsina, Joleik Nordmann, Christian Brahms, John C. Travers, Matteo Lucchini, Rocío Borrego-Varillas, Flavia Aleotti, Artur Nenov, Maurizio Reduzzi, Mauro Nisoli

**Affiliations:** 1 Dipartimento di Fisica, Politecnico di Milano, Milano, Italy; 2 Institute of Photonics & Quantum Sciences, Heriot-Watt University, Edinburgh, UK; 3 Institute of Photonics and Nanotechnologies, CNR (CNR-IFN), Milano, Italy; 4 Department of Industrial Chemistry ’Toso Montanari’, University of Bologna, Bologna, Italy

**Keywords:** Attosecond science, Atomic and molecular physics

## Abstract

Understanding ultrafast molecular dynamics requires experimental tools capable of capturing coupled electronic and nuclear motion on their natural timescales. Here, we introduce a novel UV-XUV pump-probe beamline that achieves a temporal resolution of approximately 3 fs, which significantly surpasses the precision available in conventional time-resolved photoelectron spectroscopy (trPES) setups. This performance is enabled by the synergistic synchronization of ultrashort tunable UV pump pulses, generated via resonant dispersive-wave emission in gas-filled hollow capillary fibers, with attosecond XUV probe pulses. We demonstrate the power of this approach by applying it to the study of photoexcited acetylacetone. Our approach distinctly resolves two sequential passages through the S_2_/S_1_ conical intersection (CI), revealing dynamical features previously inaccessible to trPES. By establishing a new level of temporal precision in trPES, this beamline opens a new regime for the time-domain observation of coupled electronic-nuclear wavepacket evolution in complex molecular systems, which is pivotal for advancing ultrafast photochemistry and molecular quantum control.

## Abstract (web summary)

A novel UV-XUV pump-probe beamline is introduced, enabling time-resolved photoelectron spectroscopy with 3-femtosecond temporal resolution. This performance is achieved through synchronization of tunable ultrashort UV pump pulses with attosecond XUV probes, establishing a new regime for observing coupled electronic-nuclear dynamics in molecular systems.

## Introduction

Ultraviolet-photon-induced electron dynamics lie at the heart of many fundamental processes, shaping phenomena that span atmospheric chemistry, biological function, and advanced materials science^1^. In this context, ultrashort few-cycle laser pulses in the Deep Ultraviolet (DUV, 200–300 nm) and Ultraviolet (UV, 300–400 nm) spectral regions provide exceptionally powerful phototriggers. They are ideally suited for pump-probe experiments designed to capture and interrogate these rapid, transient events with extreme temporal precision. To unravel how UV light triggers complex chemical and biological transformations, one must probe the earliest moments after photoexcitation, when the system's fate is decided. At these ultrafast timescales, the motion of electrons and nuclei becomes intricately coupled, giving rise to a strongly nonadiabatic behavior. Transitions such as conical intersections (CIs) can unfold within only a few femtoseconds^2,3^, swiftly redirecting the energy flow and steering the system toward specific reaction pathways. Capturing these fleeting events demands ultrashort UV pulses capable of resolving the fundamental steps that govern photochemical and photobiological reactivity.

Generating UV pulses with few-femtosecond durations remains challenging. Gas-based third-harmonic generation has produced sub-3-fs^4^ and even sub-2-fs DUV pulses^5,6^, but with low conversion efficiency (below 10^−3^) and limited wavelength tunability. A major breakthrough in the generation of ultrashort UV pulses was the demonstration that gas-filled photonic crystal fibers can generate UV radiation via resonant dispersive wave (RDW) emission^7–10^. Their weak anomalous waveguide dispersion allows precise tuning of the zero-dispersion wavelength and nonlinearity through gas pressure and composition, enabling broadband and efficient frequency conversion. Higher pulse energies and continuous tunability in a broad spectral range can be achieved by extending this concept to gas-filled hollow capillary fibers (HCFs)^11^. This approach provides efficient, widely tunable ultrashort pulses from the vacuum-ultraviolet (VUV) to the near-infrared (NIR), and represents a significant advance for ultrafast spectroscopy and nonlinear optics. Multiple works have recently reported on the temporal characterization of ultrashort RDW pulses^12–15^.

In parallel, RDW-generated pulses have recently been employed in a range of cutting-edge techniques, including transient reflection spectroscopy^16^, time-resolved X-ray absorption spectroscopy^17^, and time-resolved photoelectron spectroscopy (trPES)^2,18^. In trPES, the photoexcited wavepacket of the system under investigation is projected onto its cationic manifold by valence ionization, induced by interaction with a second light pulse acting as the probe, typically in the vacuum- and extreme-ultraviolet (VUV/XUV) spectral regions. The resulting photoelectrons encode detailed information on the evolving electronic and nuclear dynamics, providing a direct window into the nonequilibrium behavior of the system. The use of VUV/XUV probe pulses enables non-invasive interrogation of the pump-induced dynamics. Because these pulses typically operate in the linear or single-photon absorption regime, they introduce only minimal perturbation to the system under investigation. As a result, the evolving electronic structure and transient states can be monitored without substantially altering the underlying dynamics. Moreover, accessing higher photon-energy ranges widens the observational window, thereby allowing the detection of additional spectral features and potentially revealing signatures of ground-state dynamics as well.

To maintain energy resolution, the probe pulse in standard trPES implementations is characterized by a narrow spectrum. A variety of sources have been successfully implemented to generate such VUV and XUV pulses, including Four-Wave Mixing (FWM) in gas-filled capillaries^19,20^, monochromatized High-Order Harmonic Generation (HHG)^21,22^ and Free-Electron Lasers (FELs)^23,24^. However, a fundamental limitation of these established techniques is that the requirement for monochromaticity limits the pulse duration to  ~ 10 fs, thus limiting the achievable temporal resolution in conventional trPES measurements, effectively preventing the investigation of ultrafast dynamics that unfold on sub-10-fs timescales.

Here, we describe the development and application of a novel attosecond beamline specifically designed for high-time-resolution trPES studies in gas-phase molecular samples. The key innovation lies in combining ultrashort DUV/UV pump pulses generated via the RDW mechanism with XUV attosecond probe pulses. The synchronized delivery of these two pulses enables trPES measurements with superior temporal resolution. The overall instrument response function (IRF), a direct measure of the temporal resolution, was characterized using UV-XUV sideband analysis in a noble gas. We demonstrate a record-setting temporal resolution as low as 3.2 ± 0.2 fs. This excellent temporal resolution significantly surpasses the limitations of conventional femtosecond trPES systems, opening a new frontier for resolving dynamics on the intrinsic timescale of electronic motion.

We highlight the capabilities of this approach by applying it to the archetypal system of Acetylacetone (AcAc) following UV photoexcitation. Leveraging the  ~ 3-fs temporal resolution, we successfully resolved the ultrafast IC dynamics unfolding within the first tens of femtoseconds. The measurements provide unprecedented detailed insights into the ultrafast relaxation processes triggered by the UV pump pulse. The combined features of spectral tunability and ultra-high temporal resolution represent a substantial step forward in the capabilities of gas-phase trPES. The ability to precisely tune the DUV/UV pump pulse wavelength substantially increases the class of molecules amenable to investigation by matching the excitation energy to specific absorption bands. Furthermore, this tunability facilitates wavelength-dependent excitation studies, allowing for the targeted preparation and detailed understanding of the dynamics originating from higher-lying excited states, which is particularly crucial for exploring phenomena such as anti-Kasha photochemistry^25,26^. Moreover, the broad spectral bandwidth of the RDW UV pump not only improves temporal resolution, but also enables the coherent excitation of multiple nearby electronic states and of the associated vibronic manifold. This is a crucial aspect for future ultrafast spectroscopy applications, because it allows access to non-stationary quantum states in which electronic and nuclear motion are coherently coupled from the earliest stages of the dynamics. In this respect, such pulses are highly relevant for emerging attochemistry-oriented strategies^27^, where the preparation and probing of coherent electronic superpositions with large bandwidth may provide a route to influence photochemical evolution by acting directly on the initial electronic motion, before substantial decoherence or nuclear rearrangement takes place.

## Results

### UV pump - XUV probe trPES beamline

**Fig. 1:**
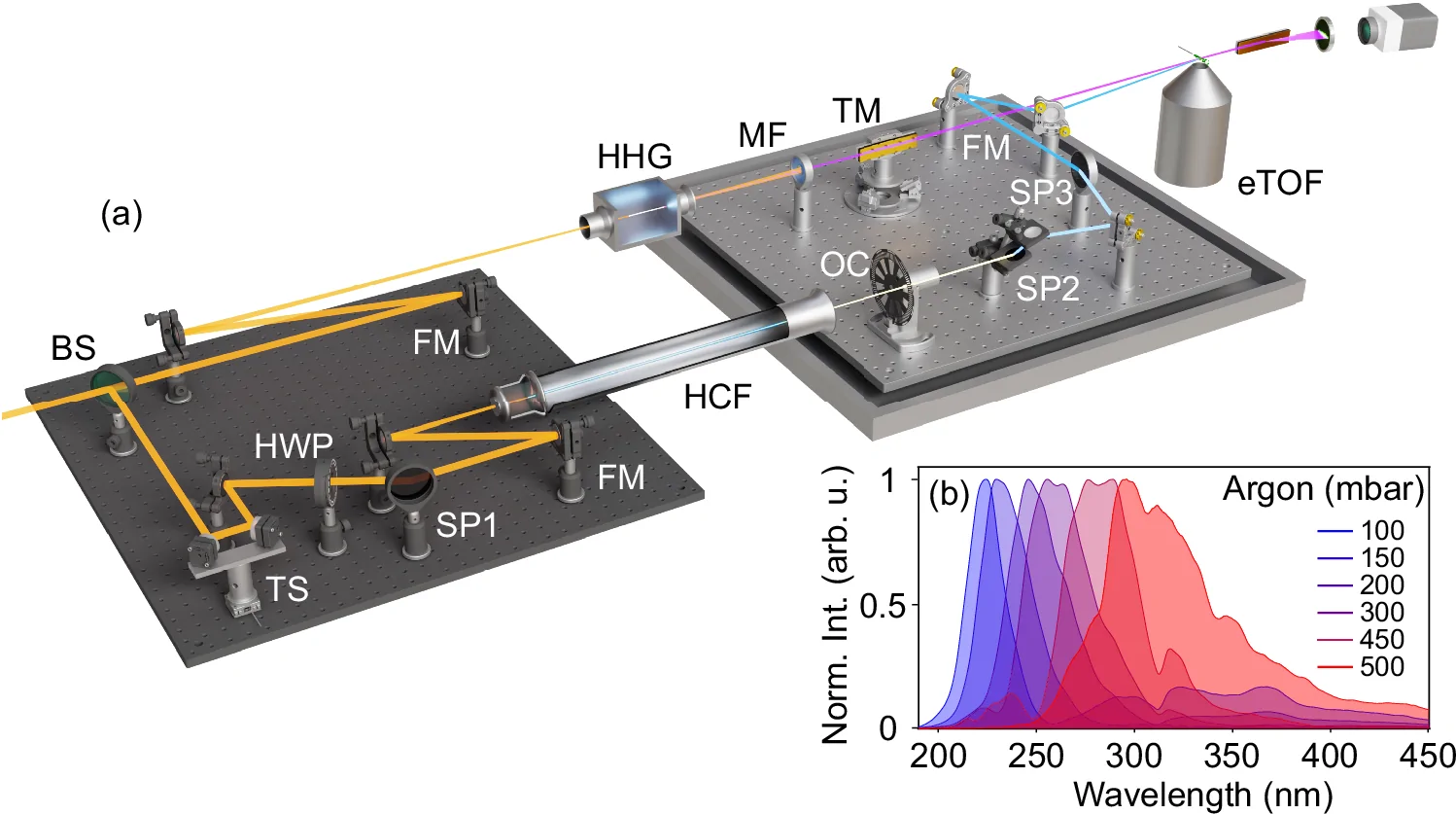
UV-pump/XUV-probe experimental setup. **a** The driving VIS-NIR beam is shown in orange, the XUV beam (produced by HHG in a gas cell) in violet, and the DUV/UV beam in light blue. BS denotes a broadband beam splitter with 20% reflectivity. The pump-probe delay is controlled using a retroreflector mounted on a nanometer-precision translation stage (TS). HWP indicates a half-wave plate, while SP1, SP2, and SP3 are silicon plates used for attenuation and polarization control. FM are the focusing mirrors for HHG and RDW generation. HCF is the hollow-capillary fiber for RDW generation. OC is an optical chopper. MF represents the metal filter employed to block the driving VIS-NIR beam. TM is the toroidal mirror used to focus the attosecond pulses into the interaction region. The photoelectrons are detected using an electron time-of-flight (eTOF) spectrometer. **b** Tunability of the UV pump spectrum, showing the normalized spectral intensity as a function of wavelength for different argon pressures at the input of the HCF.

The experiment, described in detail in the Methods section and sketched in Fig. 1a, is driven by a Ti:sapphire laser system operating at 800 nm, with 25-fs pulse duration and a 1-kHz repetition rate. Pulse compression to 4 fs is achieved using a HCF setup^28,29^, followed by dispersion compensation with a sequence of chirped mirrors. A beam splitter (BS) reflects 80% of the total visible (VIS)-NIR pulse energy (1.5 mJ) for HHG in a gas cell. This HHG stage produces XUV photons with energies between 20 and 45 eV and supports the generation of isolated attosecond pulses with durations down to 180 as^30^. The remaining 20% of the 4-fs VIS-NIR driver transmitted through BS is used to generate DUV/UV pulses via RDW emission in a gas-filled HCF. The RDW stage is identical to the one detailed in ref. ^12^, where tunable DUV/UV sub-3-fs pulses with energy in the *μ*J-range were demonstrated.

Figure 1b shows the spectral tunability of the RDW pulses generated in an argon-filled capillary for optimized input pressures. The RDW peak is tunable between approximately 230 nm and 330 nm. The corresponding UV pulse energies measured in the interaction region are presented in the Supplementary Information, together with the results obtained by filling the capillary with neon: several hundred nanojoules are obtained across the full tuning range for both gases. In neon, pulse energies exceeding 1 μJ are achieved between 270 nm and 300 nm. To ensure sub-femtosecond temporal stability, the interferometer is actively stabilized using a 405-nm continuous-wave reference laser co-propagating with the UV and XUV beams (not shown in Fig. 1b), as detailed in the Supplementary Information. Two replicas of this reference beam are extracted after the interaction region and imaged onto a camera to record spatial interference fringes. The fringe phase provides a feedback signal to correct delay drifts. This stabilization suppresses the free delay drift (~ 12 fs over one hour) to a residual timing jitter with a standard deviation of  ~ 25 as.

### In-situ characterization of the temporal resolution

To extract reliable physical insights from trPES measurements, an accurate determination of the setup temporal resolution is essential. Yet, a direct in-situ characterization of the instrumental response function (IRF) for an RDW-based source, particularly at few-femtosecond durations, poses severe technical challenges. In^14^ two-photon ionization of noble gases could be exploited, given the high photon energy of the realized tunable VUV RDW source. In this work, instead, we measure the in-situ IRF through XUV-UV cross-correlation via sideband generation in noble gases^21^.

**Fig. 2:**
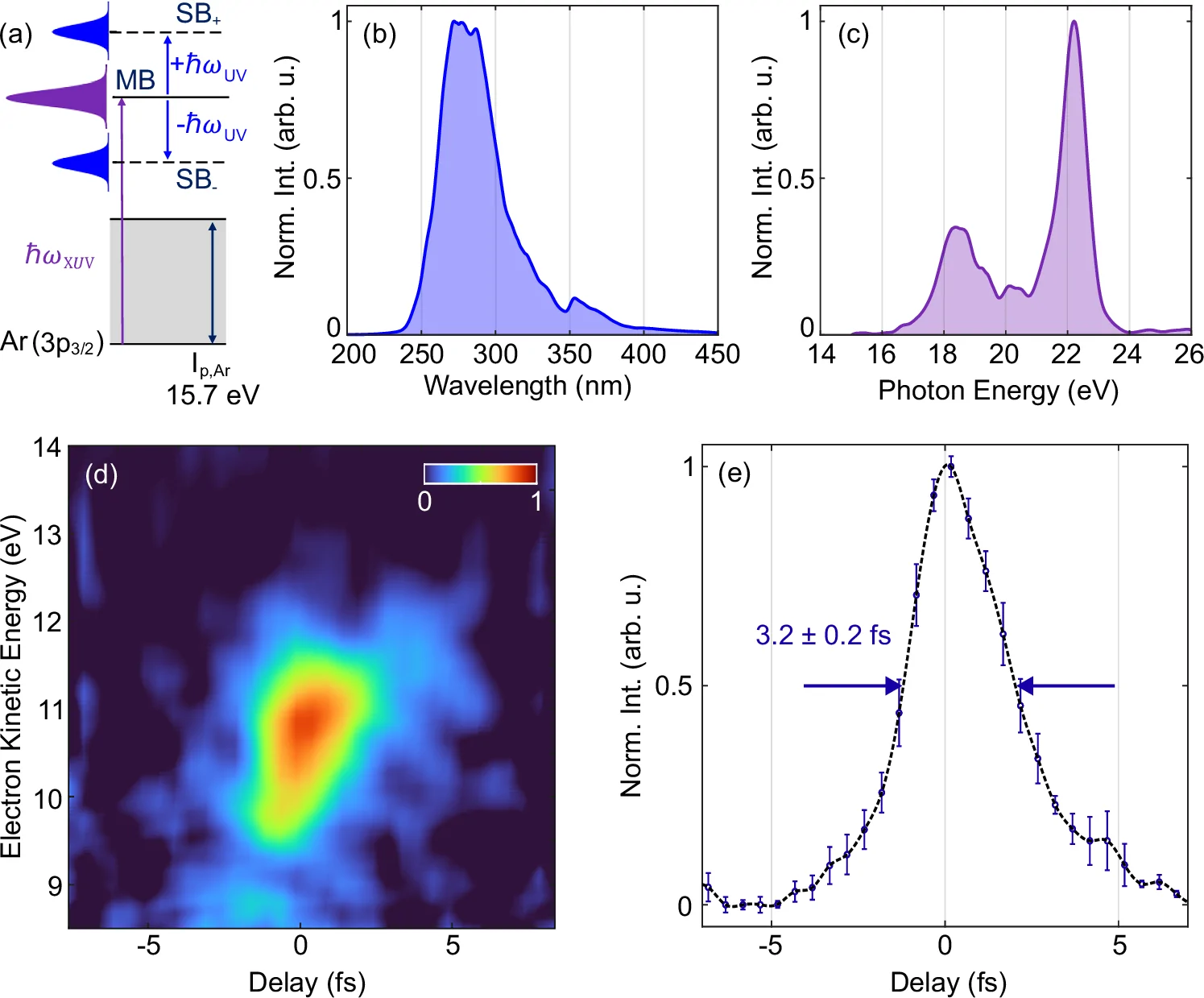
Temporal characterization of the instrumental response function. **a** Operating principle of the measurement, illustrating the processes at the basis of photoelectron mainband (MB) and sidebands (SB) formation. Spectra of the **b** UV and **c** XUV pulses used to characterize the IRF of the setup. **d** The delay-dependent photoelectron spectrum of the sideband SB_+_ signal generated in argon by the combination of the XUV and UV pulses. **e** Integrated sideband signal between 9 and 12 eV, along with the average FWHM of the measurements. Blue dots represent mean values for each delay, blue vertical bars represent  ± 1 standard error of the mean, the dashed black line is a spline interpolation between the experimental points.

In this experiment (the operating principle of which is shown schematically in Fig. 2a), direct XUV ionization of a noble-gas target produces the so-called mainband (MB) in the photoelectron spectrum. If a second pulse, in our case the UV, temporally overlaps with the XUV, the additional absorption or emission of a single UV photon becomes possible, giving rise to sideband features (SB ± ) that are shifted by  ± one UV-photon energy relative to the MB. Recording the photoelectron spectrum as a function of the XUV-UV delay yields a spectrogram that encodes the temporal structure of both pulses. When both SB_+_ and SB_-_ are recorded, iterative reconstruction algorithms can be applied to retrieve the full temporal profiles of the XUV and UV pulses without ambiguity^31,32^. Such algorithms are not applicable in case of single sideband detection; however, under the conditions of near-transform-limited Gaussian pulses and moderate UV intensity (i.e., a single SB pair), the delay-dependent signal obtained by integrating a sideband over the electron kinetic energy axis directly represents the cross-correlation between the XUV and UV pulse envelopes^33,34^. This approach enables temporal characterization even in regimes where full two-sideband reconstruction is not feasible.

The result of this procedure applied to our setup is presented in Fig. 2. Fig. 2b, c display the UV and XUV spectra, respectively. The UV pulses were produced by RDW emission in an argon-filled fiber, operated at an input pressure of 400 mbar, yielding a spectrum centered at approximately 278 nm (~ 4.45 eV). The VIS-NIR pulse energy driving RDW emission was attenuated such that the UV energy delivered to the interaction region was 200 nJ (estimated peak intensity of 7 × 10^11^ W cm^−2^). The XUV pulses were generated in xenon, with the emitted beam spectrally filtered by a 200-nm-thick Sn filter. The combined action of the XUV and UV pulses in an argon target produces the SB_+_ feature shown in Fig. 2d. We note that in the linear regime, the measured SB intensity scales as ${\omega }_{UV}^{-4}$ (where *ω*_*UV*_ is the angular frequency of the UV pulses)^35^. Therefore, a reduction of  ~ 69 times is expected when compared with similar experiments performed with 800-nm pulses of identical peak intensity. Resolving the faint SB_+_ feature requires an amplification of the eTOF signal that results in saturation for electron kinetic energies below 8.5 eV. For this reason, the mainband, as well as the SB_-_, are not shown. Integration of the SB_+_ spectrogram over electron kinetic energy (shown in Fig. 2e) and extraction of its full width at half maximum (FWHM) yield an in-situ IRF of 3.2 ± 0.2 fs for the complete setup. Further analysis and detailed comparison with simulations based on the Strong-Field Approximation (SFA) can be found in the Supplementary Information.

### Ultrafast *ππ* */*nπ* * conversion in acetylacetone

**Fig. 3:**
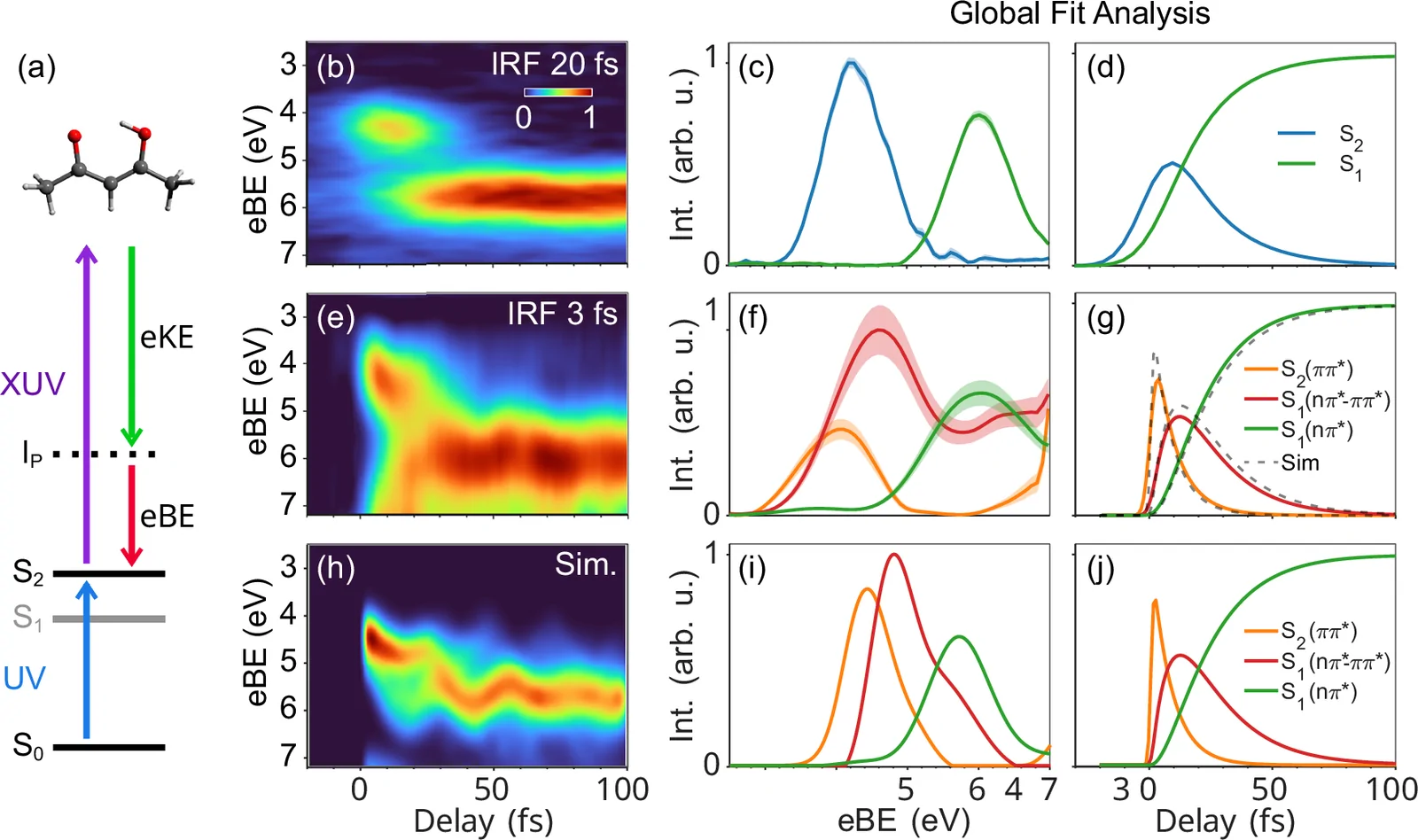
Time-resolved photoelectron spectroscopy (trPES) of acetylacetone and global-fit (GF) analysis. **a** Sketch of the experiment where a UV pump pulse excites the system and an XUV probe ionizes it; the UV-induced dynamics is tracked through the resulting electron Binding Energy (eBE), or equivalently the electron Kinetic Energy (eKE). **b–d** Experimental trPES map and global-fit analysis from ref. ^36^, showing the previously reported two-component dynamics. **e–g** Present experimental trPES data (acquired using a pump pulse with a peak wavelength of 278 nm) and GF analysis, resolving three distinct components and clear 28 fs vibrational oscillations. **h–j** CASPT2-simulated trPES data analyzed with the same GF model, reproducing the experimental spectral signatures, time constants, and oscillatory modulations. The curves shown in (**j**) are reproduced as dashed lines in (**g**). In (**c**) and (**f**) the solid lines represent the mean value, while the shaded areas extend to  ± 1 standard error of the mean. The details of the GF model used are described in the SI.

We now illustrate how the new methodology enables precision spectroscopic measurements in molecular systems, using acetylacetone (AcAc) as a representative benchmark (the molecular structure is shown in Fig. 3a). AcAc is an archetypal system exhibiting Excited State Intramolecular Hydrogen Transfer (ESIHT) dynamics and keto–enol tautomerism. In gas-phase AcAc, the enolic form, in which the intramolecular hydrogen bond gives rise to a cyclic structure, dominates^36^. AcAc is a prototypical molecular system for exploring excited-state nonadiabatic dynamics arising from the strong coupling between electronic and nuclear degrees of freedom. Following UV absorption, the population of a *ππ* * excited state initiates coherent ultrafast nuclear motion, which drives hydrogen transfer between the two oxygen atoms and *ππ* * → *nπ* * internal conversion through CI. Because these coupled processes occur on a few tens of femtosecond timescale, the excited-state dynamics of AcAc have been extensively investigated as a benchmark for developing and testing experimental and theoretical approaches to nonadiabatic dynamics^23,36–39^. Leveraging the markedly improved temporal resolution of the novel apparatus and the precise tuning of the RDW emission, we are now able to access dynamical features that have remained unresolved in all previous studies, thereby uncovering a substantially more detailed and accurate picture of AcAc early-time evolution.

Figure 3b serves as a useful reference for evaluating the additional and highly relevant dynamical information revealed by achieving a temporal resolution of  ~ 3 fs. The panel displays the two-dimensional (2D) trPES map of AcAc reported by Severino et al.^36^, obtained on a different UV/XUV beamline, based on a time-delay compensated monochromator for HHG, operating with a temporal resolution of  ~ 20 fs^21^. That dataset captures the main spectral signatures associated with the excited-state dynamics of this molecule: a strong photoelectron signal centered at  ~ 4.5 eV binding energy at time zero and a longer-lived feature emerging near 6 eV at positive delays. The characteristic oscillatory modulations visible around 6 eV are associated with a 1212 cm^−1^ vibrational mode (28 fs period), involving the motion of the central hydrogen atom along the ESIHT coordinate and the C-C/C-O bond-length alternation (BLA)^36^.

Figure 3e shows the 2D trPES map recorded in the present experiment using a 278-nm UV pump pulse. The UV pump and XUV probe pulses are the same used for the sideband measurement above, reported in Fig. 2b, c. The static photoelectron spectrum can be found in the Supplementary Information. It’s worth stressing that, although the pump spectrum spans several tens of nanometers in the DUV, in AcAc there is only one bright electronic state to be excited in the spectral range covered by the pump^40^. As such, the photoinduced dynamics can be compared with those excited in ref. ^36^. For a detailed comparison of the calculated population dynamics in the two scenarios (narrowband pump and broadband pump), the reader is referred to Supplementary Fig. 7. Notably, despite the substantially broader XUV probe spectrum compared to ref. ^36^, all spectral features previously observed with a 20-fs temporal resolution are clearly reproduced. The markedly improved temporal resolution uncovers dynamical structures that were entirely inaccessible before, providing a significantly sharper and more informative view of the early-time molecular response. We performed a global-fit (GF) analysis of the dataset. The GF procedure employs an oscillatory kinetic model that explicitly incorporates center-of-mass modulations of the individual spectral components, enabling a unified description of both population transfer and coherent nuclear motion. This model was applied not only to the experimental trPES data, but also to the CASPT2-simulated trPES signals shown in Fig. 3h. Performing the fit on both datasets ensures a consistent extraction of the underlying dynamical parameters and facilitates a direct comparison between experiment and theory. The corresponding fit results are presented in Fig. 3f, g, i, j, and the full details of the fitting strategy and implementation are provided in the Supplementary Information.

In ref. ^36^, the authors were able to resolve only two kinetic components within the first 100 fs of the UV-initiated dynamics, namely a 23 ± 2 fs decay and a concomitant rise with the same time constant (Fig. 3c, d). In contrast, the higher temporal resolution of the present experiment allows us to disentangle three distinct dynamical contributions. First, we identify an ultrafast feature at 4 eV that decays within 8.8 ± 0.3 fs (orange curves in Fig. 3f, g). Second, we observe an intermediate component characterized by a 8.8 ± 0.3 fs rise and an 15.4 ± 0.4 fs decay, with spectral weight distributed across both the 5 and 6 eV regions (red curves). Third, we resolve a long-lived contribution centered near 6 eV characterized by a rise time of 15.4 ± 0.4 fs, which remains essentially constant beyond 100 fs (green). The combined effect of the first two components reproduces the single 23 fs decay reported in ref. ^36^, while the persistent 5.5-eV signal corresponds to the long-lived feature identified in their measurements.

The CASPT2 simulations and the corresponding GF analysis shown in Fig. 3i, j not only reproduce the main characteristics of the experimental 2D map but also match the kinetic parameters extracted from the GF analysis, yielding decay and rise constants of 8.1 fs and 17.2 fs, respectively: values in very good agreement with the experimental ones. Moreover, the oscillatory terms retrieved from the fit provide a consistent picture across both experiment and theory: in each case we extract an oscillation period of  ~ 28 fs (1212 cm^−1^). The remaining discrepancies between simulated and experimental trPES signals, most notably in binding-energy positions and relative intensities (Fig. 3f, i), can be attributed to technical aspects of the simulation protocol (further details can be found in the Supplementary Information).

**Fig. 4:**
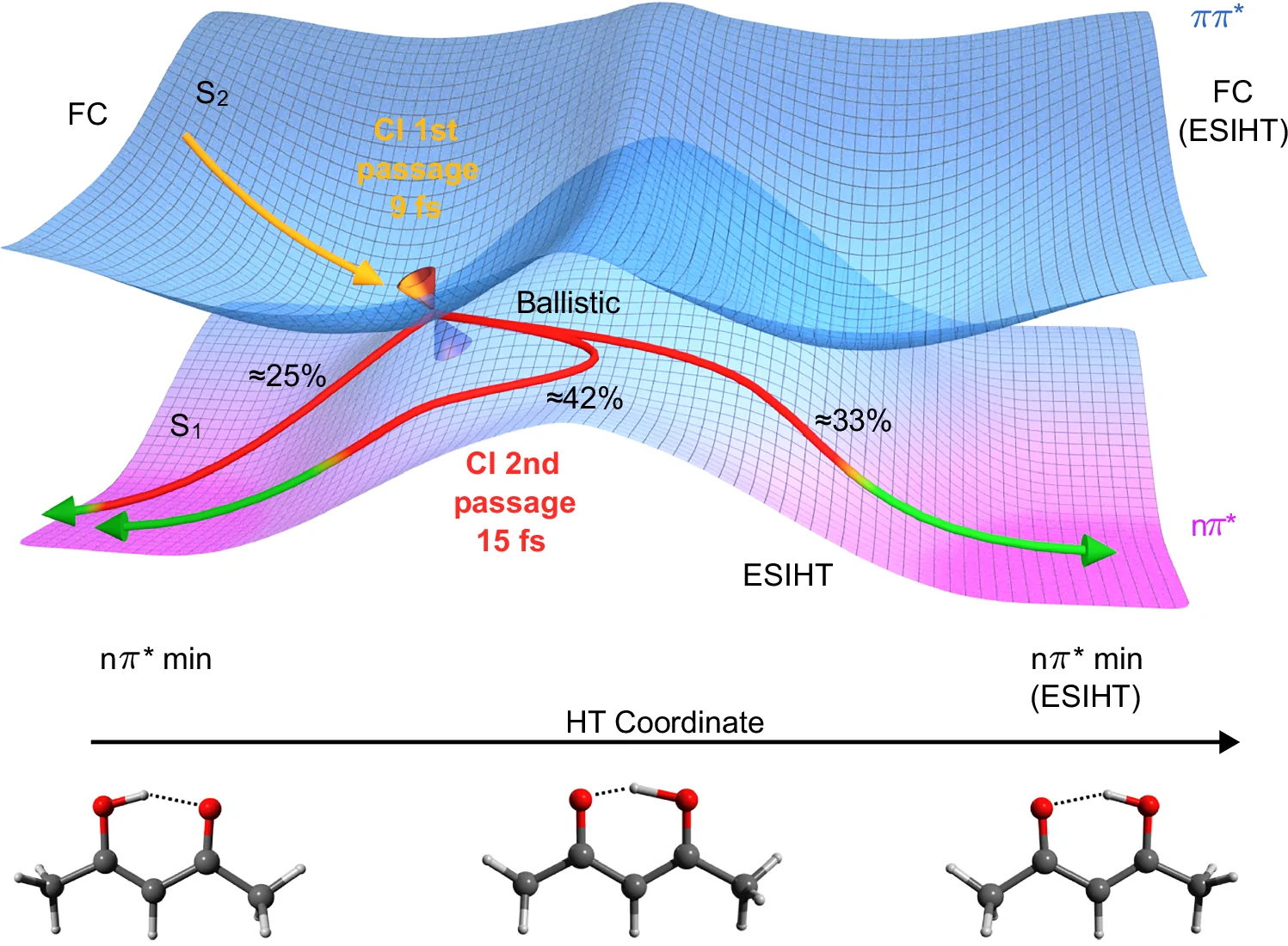
Acetylacetone short-timescales deactivation mechanism. Sketch of the potential energy surfaces (PESs) of S_1_ and S_2_ where the arrows represent characteristic trajectories. The color refers to the GF components as in Fig. 3. After the first CI passage in 9 fs, most of the trajectories retain an ππ^*^ character (blue surface), while approximately 25% of them undergoes an early change in electronic configuration assuming nπ^*^ character (pink surface). A second passage around the CI completes the electronic transition to the nπ^*^ in approximately 15 fs. Below the sketch of the PESs, the hydrogen transfer (HT) coordinate is depicted: the distance between the apical hydrogen and the oxygen atoms is reversed from left to right.

Given the excellent agreement between experiment and theory, we can assign a physically consistent interpretation to the kinetic components extracted from the GF analysis. Figure 4 shows the potential-energy surfaces (PESs) involved in the short-timescale dynamics and summarizes the UV-induced relaxation mechanisms that take place. Following excitation in the FC region, the nuclear wave packet begins to evolve on the *S*_2_ (*ππ* *) PES and reaches the CI seam between the *S*_2_ and *S*_1_ states in roughly 9 fs. This ultrafast motion can be monitored through the photoelectron signal at an electron binding energy of approximately 4 eV, which is assigned to ionization into the *D*_0_ ionic state, corresponding to the removal of the excited *π* * electron, and yielding a cation with ${[{\pi }^{1}]}^{+}$ character. This first passage through the CI leads to the rapid disappearance of the  ~4 eV signal (corresponding to the orange component in the GF analysis). Simulations suggest that this initial crossing is largely ballistic and diabatic: most trajectories retain their *ππ* * character after the transition to *S*_1_, producing the signal centered at 5 eV. The  ~ 1 eV blue-shift relative to the *D*_0_ feature reflects the fact that the wavepacket, while still retaining ππ* character, is no longer ionized from the Franck-Condon geometry. As it evolves toward lower-energy regions of the *ππ* * potential-energy surface, the vertical ionization energy changes, leading to a photoelectron feature at higher binding energy. However, approximately 25% of the trajectories undergo an early *ππ* *-to-*nπ* * change in electronic configuration due to out-of-plane vibrations, already thermally activated in the ground electronic states. This nonadiabatic event manifests (both in the experimental and simulated traces) through a contribution at 6 eV due to ionization to the ${[{n}^{1}]}^{+}$ state *D*_1_ and is already visible at early pump-probe delays. Between about 10 and 20 fs, the wave packet continues to evolve along the ESIHT coordinate in a region where the *S*_2_(*ππ* *) and *S*_1_(*nπ* *) surfaces are well separated in energy; as a result, the relative intensities of the *ππ* * and *nπ* * contributions remain nearly constant during this interval (red GF component). Within approximately 15 fs the wave packet has turned around and returns toward the intersection region. However, the sharply peaked topology of the lower potential energy surface near the CI seam forces it to move around the seam, inducing strong vibronic couplings along symmetry-breaking modes and thereby promoting efficient *ππ* * → *nπ* * population transfer: the *ππ* * component decays sharply while the *nπ* * signal increases correspondingly, thereby completing the electronic transition (green GF component). The decomposed CASPT2-simulated trPES maps provided in the Supplementary Information (see Supplementary Fig. 8) support this interpretation: the *S*_2_ contribution is fully depleted after the first CI passage at  ~ 8 fs; the *nπ* * signal at 6 eV appears by  ~ 8 fs and evolves steadily over the next 10 fs before undergoing again a sharp increase around  ~ 17 fs.

A more detailed trajectory-based analysis of the CASPT2 dynamics further reveals that the distinction between full and aborted hydrogen transfer is governed primarily by the initial momentum along the ESIHT coordinate, rather than by the topology of the potential surfaces, which explains the absence of distinct spectral signatures for these two subgroups. The simulated 2D maps for the two trajectory classes, shown in the Supplementary Information, confirm that their photoelectron signatures are nearly indistinguishable, consistently reproducing the same timing and relative amplitude of the *ππ* * and *nπ* * components. As discussed in the Supplementary Information, complete O − H ⋯ O  →  O ⋯ H − O transfer is obtained in 33% of the cases.

Overall, these results demonstrate that the new beamline provides the sensitivity and temporal precision required to resolve population transfer processes occurring on sub-10-fs timescales and to track coherent vibronic motion in complex molecular systems. The markedly improved temporal resolution of our setup does far more than refine previously reported dynamics: it uncovers key ultrafast processes that were completely beyond the reach of earlier experiments. Most notably, it allows us to directly resolve the two successive passages through the conical intersection, critical features of the excited-state landscape that had remained experimentally invisible until now. The resulting picture is markedly more complete, internally consistent, and physically transparent than what could be achieved with previous trPES implementations, underscoring the substantial leap in capability enabled by our approach.

## Discussion

We have developed a novel UV-XUV pump-probe beamline that achieves few-femtosecond temporal resolution and enables fundamentally new classes of ultrafast measurements in gas-phase molecular systems. By combining ultrashort DUV/UV pump pulses, generated through RDW emission in gas-filled HCFs, with attosecond XUV probe pulses, the setup achieves an in-situ instrument response of approximately 3 fs. This represents a substantial advance over conventional femtosecond trPES platforms, overcoming long-standing limitations in temporal resolution.

The application to acetylacetone demonstrates the scientific impact of these capabilities. Whereas earlier 20-fs trPES measurements could resolve only two kinetic components, the present setup clearly disentangles three distinct population-dynamical channels and directly reveals two sequential passages through the S_2_/S_1_ conical intersection: features that had remained entirely inaccessible until now. The excellent agreement with CASPT2 simulations further validates the mechanistic interpretation and demonstrates the beamline's ability to capture nonadiabatic dynamics with unprecedented precision.

Importantly, acetylacetone should be viewed as a prototypical case rather than a special one. Because similarly rapid electronic and vibronic processes are expected to occur across a broad class of polyatomic molecules, the enhanced time resolution demonstrated here substantially broadens the range of phenomena that can now be experimentally accessed. The ability to disentangle distinct ultrafast population-dynamical channels opens the door to uncovering mechanisms that have remained hidden so far and enables systematic exploration of nonadiabatic processes in many molecular systems that were previously beyond reach.

In this broader context, the UV-XUV beamline establishes a new standard for gas-phase time-resolved photoelectron spectroscopy. Its unique combination of ultrashort pulse duration, broad wavelength tunability, and attosecond-class synchronization opens the door to a wide range of advanced studies, from wavelength-selective excitation of higher-lying states to the preparation and interrogation of coherent electronic wavepackets relevant to attochemistry. By enabling the observation of molecular dynamics on the fastest electronic and vibronic timescales, the beamline provides a powerful and versatile tool for advancing fundamental understanding in ultrafast photophysics and photochemistry.

## Methods

### Experimental setup

The experiment is driven by a carrier-envelope-phase (CEP)-stabilized (the CEP standard deviation is  < 300 mrad) Ti:sapphire laser system (Legend DUO HE+, Coherent) operating at 800 nm, with 25-fs pulse duration and 1-kHz repetition rate. From the total pulse energy of 10 mJ, 6 mJ are directed to a post-compression stage. This stage consists of spectral broadening in a gas-filled HCF^28,29^, followed by dispersion compensation using a sequence of chirped mirrors.

Due to the high pulse energy, a 3-m-long stretched HCF^41^, with a core diameter of 450 *μ*m, is used in an increasing gas pressure gradient configuration^42^. Helium has been used as nonlinear medium, maintaining a gas pressure of 10^−1^ mbar at the fiber input and 1.8 bar at its output. This configuration is designed to avoid undesired nonlinear effects, such as self-focusing, filamentation, and gas ionization at the input of the HCF, ensuring a purely SPM-driven spectral broadening^43,44^. A two-point active stabilization system is used to set and maintain the laser beam pointing along the fiber, which has a total transmission of approximately 67%. The laser beam at the fiber output is collimated and then recompressed with a set of chirped mirrors (PC1332, Ultrafast Innovation). The time duration of the pulses used to drive the RDW emission and HHG processes has been characterized in-situ by a second harmonic D-Scan apparatus^45,46^, demonstrating a FWHM time duration of 4 fs.

After the chirped mirrors, 80% of the total (1.5 mJ) visible/near-infrared (VIS-NIR) energy is reflected by a beam splitter (BS) and used to generate XUV attosecond pulses via HHG in a gas cell. A metallic mask blocks the external part of the residual driving VIS-NIR beam just after the XUV generation, exploiting its larger divergence with respect to the XUV. The remaining part of the HHG driver is instead removed via a metallic filter before XUV focusing. The XUV beam is then focused into the interaction region by a gold-coated toroidal mirror. A grating-based spectrometer is positioned at the end of the beamline for HHG optimization and monitoring.

The remaining 20% of the 4-fs VIS-NIR driver transmitted through the beam splitter BS is used to generate DUV/UV pulses via RDW emission in a gas-filled HCF. Before being injected into the HCF, the VIS-NIR beam is appropriately delayed, and its energy is finely adjusted to optimize RDW generation. Attenuation is controlled using an ultrabroadband half-wave plate (B. Halle) in combination with a silicon plate set at the Brewster angle for 800 nm (74.9^∘^). This arrangement functions as a broadband polarizer while preserving the pulse bandwidth and avoiding significant dispersion. The conditioned beam is then focused into a rigid HCF with a 160-μm core diameter and a length of 0.6 m, where RDW emission occurs. The beam pointing at the fiber input is actively stabilized, and the maximum available energy at the fiber input is 160 μJ. The capillary is directly interfaced to the vacuum chambers of the beamline, and it is filled with a decreasing gas pressure gradient^12,47^. A differential pumping stage between the fiber end and the vacuum chamber prevents the overload of the beamline vacuum system.

The beam at the HCF output is partially collimated by an aluminum concave mirror with focal length *f* = 1 m. Spectral separation of the UV content of the beam from the residual driving VIS-NIR is performed by a pair of silicon plates at Brewster angle for both S- and P-polarization of the VIS-NIR beam. We verified that both reflections are required in our implementation to efficiently extinguish the VIS-IR light and let only the desired DUV/UV radiation propagate towards the sample. This solution is well established for dispersionless separation of UV pulses from the VIS-NIR driving field with reported VIS-NIR suppression of 2–3 orders of magnitude^4,5,12,48^. Although this method preserves the entire broad bandwidth of the tuneable RDW pulses, it becomes inefficient for increasing wavelengths. In fact, the total reflectivity of the two silicon plates decreases from approximately 42% at 210 nm to almost 4% at 400 nm. Exploitation of dichroic mirrors would, of course, be a compelling alternative. However, to the best of our knowledge, ultrabroadband, group delay dispersion (GDD)-controlled dichroic mirrors in the UV/DUV are not yet available. A compromise can be made, as done in ref. ^17^, accepting to operate at a fixed wavelength (typically the third harmonic of the driving radiation, at 270 nm).

After spectral filtering, the UV pulses are directed onto a second concave mirror with a focal length *f* = 0.6 m, which focuses the UV beam into the interaction region, ensuring that its focal plane coincides with that of the XUV beam. Before recombination, a small fraction of the UV beam is extracted for diagnostic purposes, allowing direct measurement of the on-target RDW spectrum and UV pulse energy.

Supplementary Fig. 2 shows the UV beam in the case of a pulse with central wavelength at 250 nm. The 2D spatial distribution and beam size measured over a 3-cm region around the recombination focus with the XUV reveal an astigmatic profile. Despite this aberration, the beam forms a least-confusion focal spot with 1/e^2^ diameters of 90 and 95 μm. Accounting for a time duration FWHM of the UV pulses of 3 fs^12^, the maximum estimated UV peak intensity, obtained in neon, is of 1.1 × 10^13^ W/cm^2^.

The UV and XUV pulses are noncollinearly focused onto the gas-phase molecular target within the interaction region at a recombination angle smaller than 0.3^∘^. The spatial overlap between the two beams is performed by optimizing the transmission of both of them through a pinhole with a diameter of 50 μm that can be placed in the interaction point. The presence of a chopper on the UV beam path enables the acquisition of XUV-only photoelectron signals during the time-resolved measurements, switching on and off the UV pulses. Photoelectrons generated by the UV-XUV interaction are detected using a time-of-flight (TOF) spectrometer (Stefan Kaesdorf ETF 15).

The gas sample is delivered to the UV-XUV interaction region via a needle mounted on a three-axis manipulator, enabling optimization of the gas jet position relative to the spatially and temporally overlapping pump-probe beams. Gas pressure in the experimental region is precisely controlled via a needle valve. Both the sample holder and delivery line can be heated to regulate temperature and enhance vapor delivery when samples do not exhibit sufficient vapor pressure at room temperature.

## Supplementary information

Supplementary Information

Transparent Peer Review file

## Data Availability

The experimental data, the results of the simulations and the results of the data analysis are available via Zenodo at https://doi.org/10.5281/zenodo.20811908 (ref. ^49^).

## Code availability

COBRAMM is available on GitLab at https://gitlab.com/cobrammgroup/cobramm. OpenMOLCAS (v. 23.10) is available at https://gitlab.com/Molcas/OpenMolcas.git. Gaussian (v. 16) is available from Gaussian Inc., subject to licensing restrictions.
