# Supplementary material for: 3-fs-resolved UV/XUV photoelectron spectroscopy in the gas phase
Source: Nat Commun. 2026 Aug 19;17:9912. doi: 10.1038/s41467-026-76602-4 (PMC13582955; doi:10.1038/s41467-026-76602-4)
Supplement: Supplementary file 1 [file 41467_2026_76602_MOESM1_ESM.pdf]

Supplementary information for: 3-fs-resolved UV/XUV photoelectron spectroscopy in the gas phase

Marta Pini[1†], Stefano Severino[1†], Lorenzo Mai[1†], Marta Ortolan[1], Daniele Mocci[1], Lorenzo Colaizzi[1], Nikoleta Kotsina[2], Joleik Nordmann[2], Christian Brahms[2], John C. Travers[2], Matteo Lucchini[1,3], Rocío Borrego-Varillas[3], Flavia Aleotti[4], Artur Nenov[4], Maurizio Reduzzi[1*], Mauro Nisoli[1,3*]

[1]Dipartimento di Fisica, Politecnico di Milano, Piazza Leonardo da Vinci 32, Milano, 20133, Italy.
[2]Institute of Photonics Quantum Sciences, Heriot-Watt University, Edinburgh, UK.
[3]Institute of Photonics and Nanotechnologies, CNR (CNR-IFN), Piazza Leonardo da Vinci 32, Milano, 20133, Italy.
[4]Department of Industrial Chemistry "Toso Montanari", University of Bologna, Via Piero Gobetti, 86, Bologna, 40129, Italy, Italy.

*Corresponding author(s). E-mail(s): maurizio.reduzzi@polimi.it; mauro.nisoli@polimi.it;
†These authors contributed equally to this work.

Contents

1 Experimental details **2**
- 1.1 Spectral characteristics and energy of the UV pulses 2
- 1.2 Transform-limited duration of the UV pulses 2
- 1.3 Spatial characteristics of the UV beam 2
- 1.4 Active stabilization of the pump-probe interferometer 2

2 Instrumental response function **4**

3 trPES simulations 8
3.1 Spectroscopy simulation . . . 8
3.2 Decomposition of simulated trPES contributions . . . 9
3.3 Discrepancies between experimental and calculated trPES traces . . . 12

4 Static Photoelectron Spectrum 12

5 Global fit reconstructions 12

1 Experimental details

1.1 Spectral characteristics and energy of the UV pulses

Supplementary Fig. 1(a)-(b) show the spectral tunability of the RDW pulses generated in a 0.6-m-long HCF with a 160μm inner radius, filled with neon and argon, respectively, for different values of the input gas pressure, under optimized conditions. When neon is used, the peak of the UV pulses spans the wavelength range from approximately 230 nm to 330 nm. With argon, the tunability extends from about 220 nm to 300 nm. Supplementary Fig. 1(c)-(d) present the corresponding UV pulse energies measured in the interaction region.

1.2 Transform-limited duration of the UV pulses

Table 1 reports the central wavelength and the transform-limited durations of the UV pulses corresponding to the spectra shown in Supplementary Fig. 1, for different gas pressures at the entrance of the HCF used to generate the RDW emission.

1.3 Spatial characteristics of the UV beam

The beam profile of the UV pulses with spectrum peaked at 250 nm is reported in Supplementary Fig. 2. The 2D spatial distribution and the spot size of the UV along the two principal orthogonal axes in a range of 3 cm around its focal spot where it recombines with the XUV, show that the beam suffers from astigmatic aberration, still guaranteeing a focal spot in the least confusion position with 1/e^2 radii of 45 μm and 48 μm.

1.4 Active stabilization of the pump-probe interferometer

Active stabilization of the interferometer is required to perform time-resolved measurements with sub-fs to few-fs temporal resolution. For this purpose, a continuous wave (CW) monochromatic diode laser at 405 nm (Cobolt 08-01 NLD) is injected in the interferometer from the back of the beamsplitter BS, and co-propagates with the laser beams in the UV and XUV beam paths. On the XUV arm, the CW laser propagates through the transparent glass mount of the metallic filter used to clean the XUV from the residual driving visible-near infrared (VIS-NIR) beam, due to the larger UV divergence with respect to the XUV. The residual VIS-NIR co-propagating with the CW laser is filtered out by a 3-nm-narrow band pass filter around the wavelength of the CW laser, not intercepting the XUV radiation. The two replicas of the

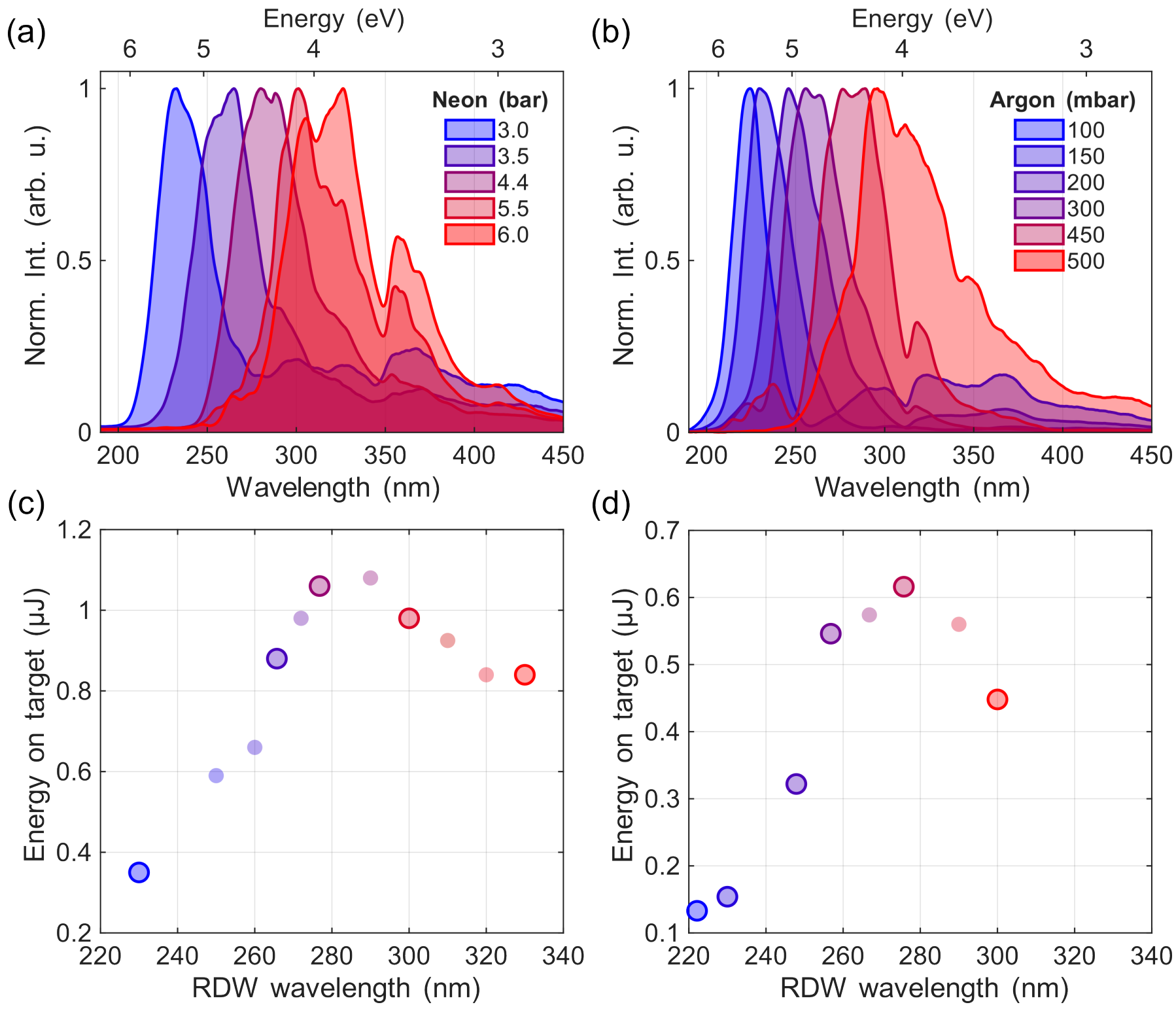

Supplementary Fig. 1 | Tunable RDW emission. Measured RDW spectra from a 0.6-m-long HCF with 160 μm core radius filled with neon (a) and argon (b), recorded for different gas pressures at the fiber input. Corresponding measured pulse energies on target for neon (c) and argon (d) are shown as a function of the RDW central wavelength. Panels (c) and (d) include a larger dataset than shown in (a) and (b), where only a subset of spectra is displayed for clarity; points corresponding to the displayed spectra are highlighted with larger markers and outlines.

CW laser are simultaneously reflected by a pick-off mirror placed on the beam paths after the interaction region, and recombines outside the vacuum system on a CMOS camera A synchronized chopper is placed before the camera to remove the residual VIS-NIR radiation, and the spatial interference fringes in the blue are detected. The phase shift of the interference pattern, directly linked to the pump-probe delay shift, is used to feedback the delay-stage position, compensating for drifts. The left panel of Supplementary Fig. 3 shows the correction applied to the delay stage to maintain a constant delay between the pump and probe pulses over time (blue dots). This correction can be interpreted as a measure of the free drift of the delay, and it amounts to approximately 12 fs over one hour of measurements. The same figure also displays the behavior of the stabilized delay over the same time scale (violet dots). The Gaussian distribution of the residual error in the imposed delay, shown in the right panel

Gas	Pressure (bar)	RDW peak (nm)	FTL (fs)
Neon	3.0	232	2.5
	3.5	265	2.5
	4.4	275	2.1
	5.5	301	2.2
	6.0	327	1.9
Argon	0.100	224	3.1
	0.150	230	2.5
	0.200	246	2.4
	0.300	256	2.2
	0.450	275	2.0
	0.500	299	1.7

Supplementary Table 1 | Peak wavelength of the UV pulses, Fourier Transform Limited duration (FTL), noble gas used and its pressure at the input of the HCF for the generation of the UV pulses corresponding to the spectra shown in Supplementary Fig. 1.

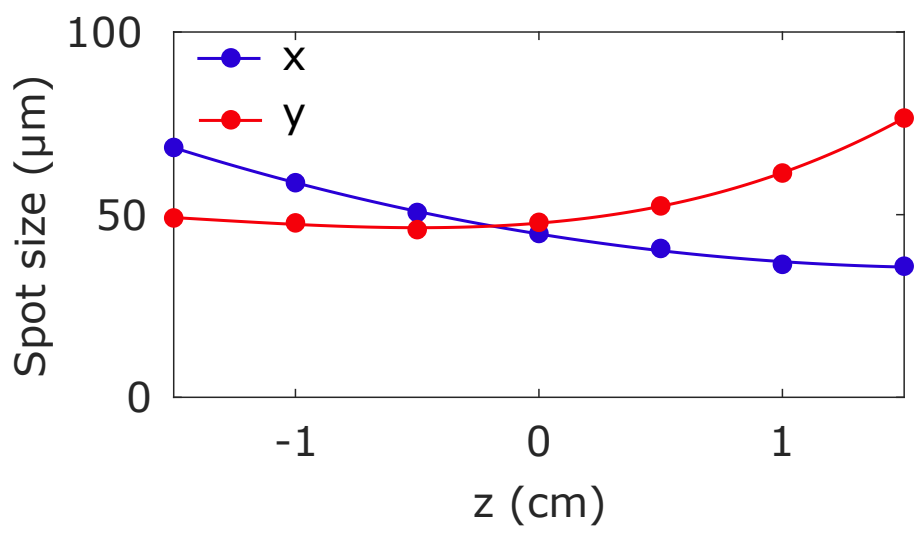

Supplementary Fig. 2 | **Spatial characteristics of the UV beam.** Spot size of the UV beam (250-nm central wavelength) along the x (blue curve) and y (red curve) axis, orthogonal to the propagation direction z.

of Supplementary Fig. 3 exhibits a full width at half maximum of approximately 58 as, corresponding to a residual standard deviation of 25 as.

2 Instrumental response function

We performed Strong-Field Approximation (SFA) calculations following the formalism described by [1], using as input the experimental UV and XUV spectra reported in Fig. 2(b) and 2(c), respectively, of the main manuscript. In the simulations, no spectral phase was assigned to the XUV pulse. As discussed below, this approximation does not represent a significant limitation for the present analysis. To reproduce the experimental conditions under which the SB+ spectrogram shown in Fig. 2(d) was measured,

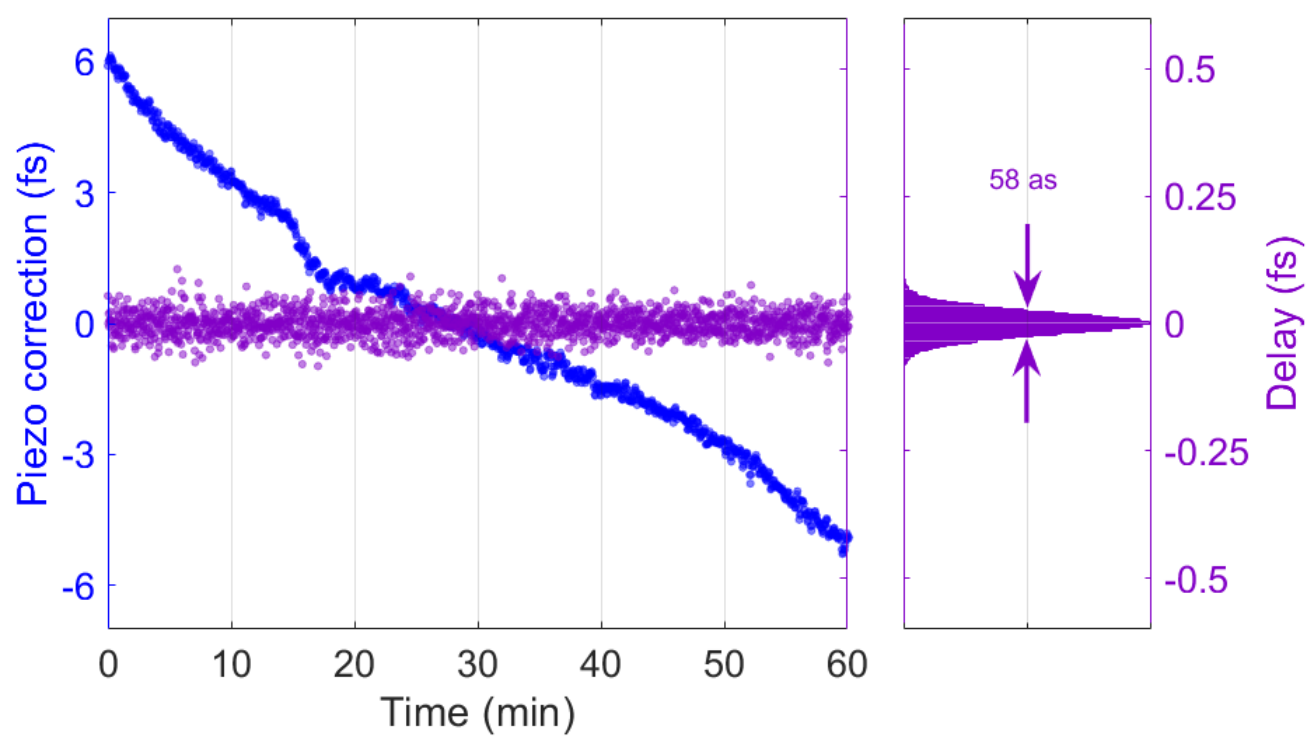

Supplementary Fig. 3 | Active stabilization of the pump-probe interferometer Left panel: Correction applied to the delay stage to maintain a constant delay between the pump and probe pulses over time (blue dots) and residual interferometer instabilities with active stabilization (violet dots). Right panel: The histogram of the residual interferometer instabilites, revealing a FWHM of 58 as, corresponding to a standard deviation of about 25 as.

the UV peak intensity was set to 7×10^{11} W cm^{-2}. In addition, a parabolic spectral phase was applied to the UV pulse, corresponding to a group-delay dispersion (GDD) of 1.5 fs^2. Under these conditions, the UV pulse duration becomes 3.08 fs FWHM, in very good agreement with the pulse duration expected from our previous characterization [2]. The experimental and calculated delay-dependent photoelectron spectrum of

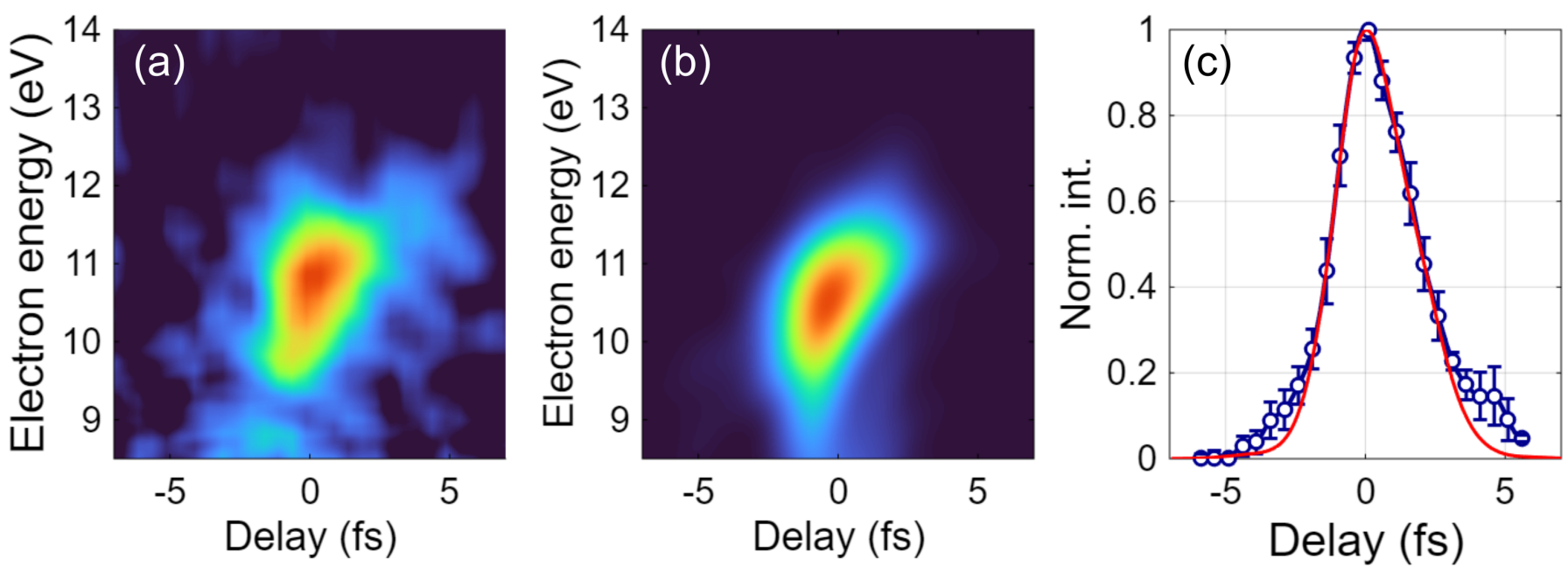

Supplementary Fig. 4 | Experimental and simulated SB+ photoelectron spectra. (a) Experimental spectrum, identical to that shown in Fig. 2(d) of the main text. (b) Calculated spectrum. (c) Comparison of the delay-dependent SB+ signal extracted from experiment and simulation: blue open circles with error bars show the experimental data, identical to those in Fig. 2(e) of the main text, while the red solid curve shows the simulated profile, shifted by 500 as toward positive delay for clarity.

the sideband SB+ signal are shown in Supplementary Fig. 4(a) and 4(b), respectively;

the experimental data are the same as those presented in Fig. 2(d) of the main text. In Supplementary Fig. 4(c), the blue open circles with error bars represent the experimental data, identical to those shown in Fig. 2(e) of the main manuscript, whereas the red solid curve corresponds to the simulated profile (shifted by 500 as toward positive delay for clarity). Overall, the agreement between experiment and simulation is very satisfactory in all panels, and notably this is achieved without introducing any additional adjustable parameters beyond those independently determined from the experimental conditions. To elucidate the physical origin of the signal observed in

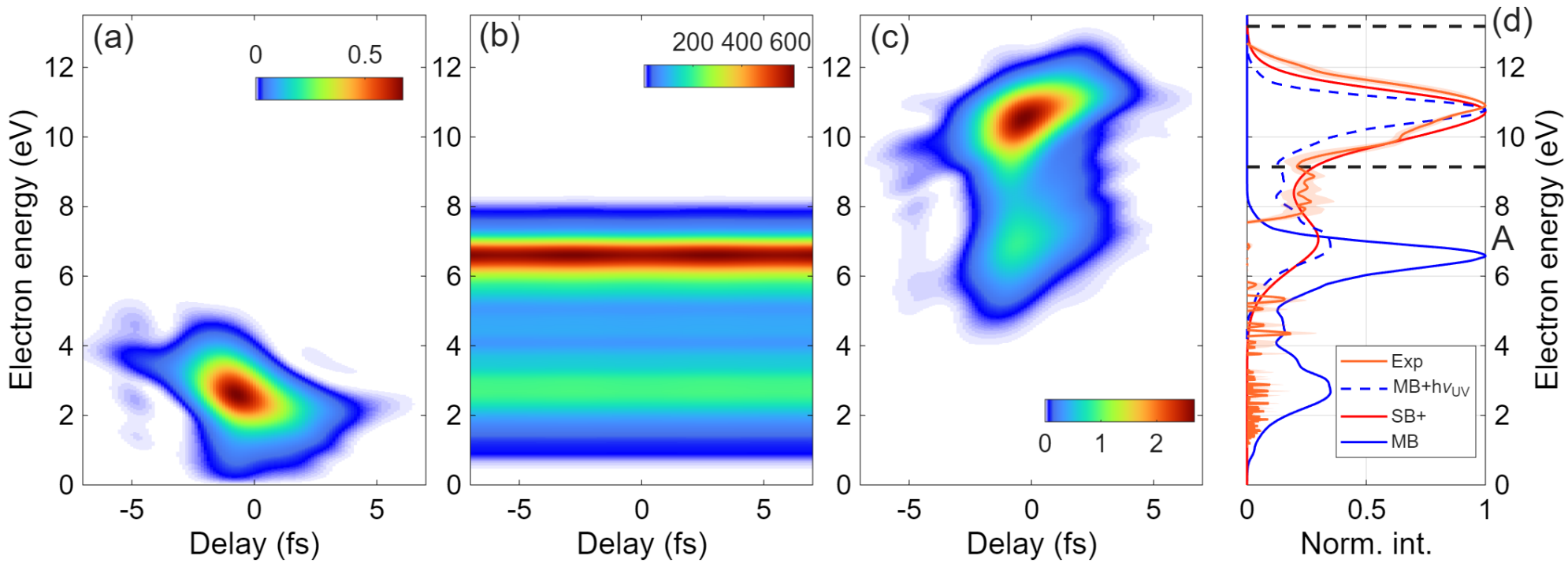

Supplementary Fig. 5 | Decomposition of the calculated spectrogram into the SB−, MB, and SB+ contributions. (a) Lower-sideband contribution (SB−), associated with absorption of one XUV photon and emission of one UV photon. (b) Main-band contribution (MB), corresponding to direct XUV ionization. (c) Upper-sideband contribution (SB+), associated with absorption of one XUV photon and absorption of one UV photon. (d) Delay-integrated spectra corresponding to panels (b) and (c), shown as solid blue and solid red curves, respectively, together with the experimental profile extracted from Supplementary Fig. 4(a) (solid orange curve with shaded area). The dashed blue curve shows the MB contribution shifted by one UV-photon energy.

Supplementary Fig. 4(a), we decomposed the calculated spectrogram according to the procedure introduced in [3]. The results are reported in Supplementary Fig. 5. Panel (a) shows the contribution associated with the process involving absorption of one XUV photon and emission of one UV photon, i.e. the SB− channel. Panels (b) and (c) display the spectrograms associated with direct XUV ionization (main band, MB) and with the first upper sideband (SB+), respectively. In Supplementary Fig. 5(d), we report the corresponding delay-integrated spectra obtained from Supplementary Fig. 5(b) (solid blue curve) and Supplementary Fig. 5(c) (solid red curve), together with the experimental profile extracted from Supplementary Fig. 4(a) (solid orange curve with shaded area). This comparison shows that the SB+ signal spectrally overlaps with the MB contribution. In the experiment, however, this overlapping region cannot be accessed because the detector saturates. To facilitate the comparison, the MB profile has been shifted upward by one UV-photon energy, yielding the dashed

blue curve in Supplementary Fig. 5(d). Its qualitative similarity to the SB+ profile (solid red curve) indicates that the spectral region of SB+ used to estimate the instrumental response function (IRF), namely the interval delimited by the horizontal black dashed lines, originates predominantly from the absorption of one XUV photon associated with the peak labeled A in Supplementary Fig. 5(d), corresponding to approximately 22 eV in the original XUV spectrum. At sufficiently low UV intensity,

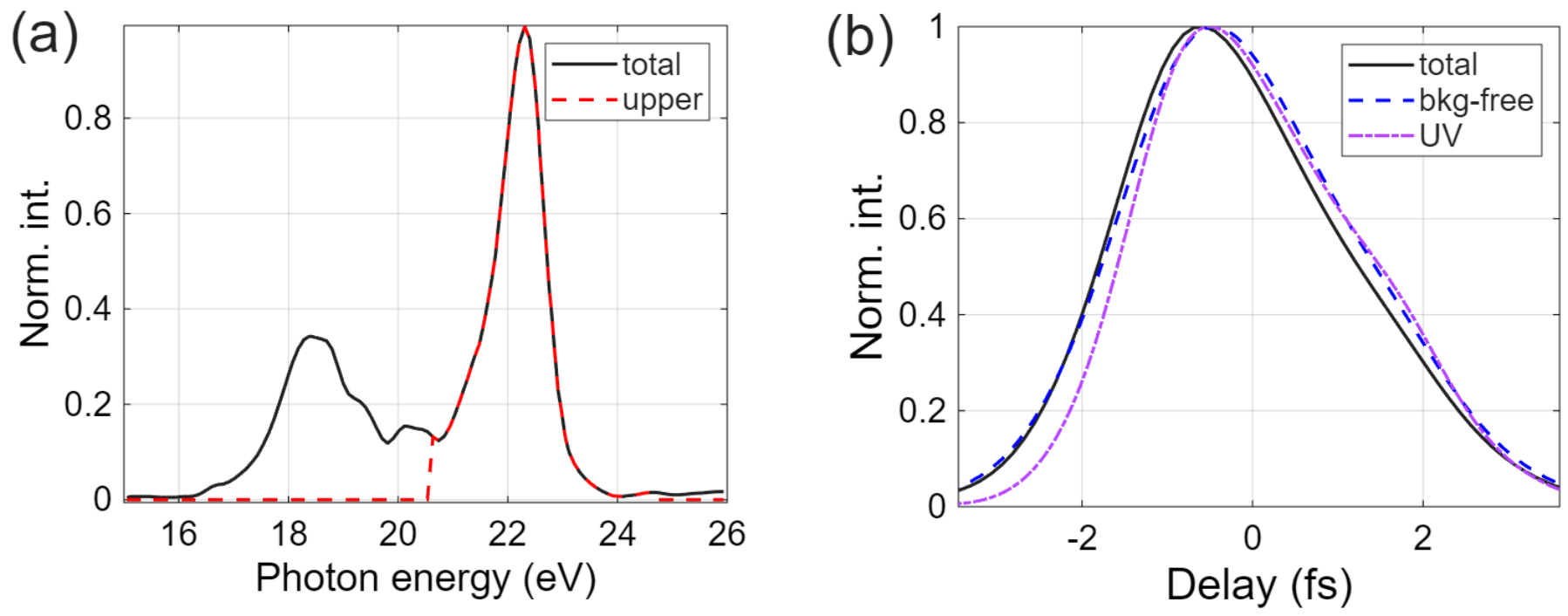

Supplementary Fig. 6 | Spectral range contributing to the visible SB+ signal and corresponding temporal traces. (a) Experimental XUV spectrum, identical to that shown in Fig. 2(c) of the main text (solid black curve), together with the portion of the XUV spectrum that gives rise to the measurable part of the SB+ signal (dashed red curve). (b) Corresponding normalized temporal profiles obtained by integrating the SB+ over the full spectral region (solid black curve) and over the background-free spectral region that is experimentally accessible (dashed blue curve), shown together with the UV pulse profile (dash-dotted purple curve).

the delay-integrated SB+ spectrogram corresponds to the convolution of the spectral intensities of the XUV and UV pulses, whereas the energy-integrated SB+ trace corresponds to the convolution of their temporal intensities [4]. In the present analysis, the energy integration used to retrieve the IRF is performed only over the upper portion of the SB+ signal, i.e. over the region associated with the dominant peak of the XUV spectrum (see Supplementary Fig. 6(a)). Therefore, only a reduced portion of the XUV spectrum contributes to the measured signal. Since this portion is spectrally narrower than the full XUV pulse, the retrieved cross-correlation width should be regarded as an upper limit to the actual one (solid black and dashed blue curves in Supplementary Fig. 6(b)). This effect is further illustrated by the transform-limited pulse durations associated with the relevant spectral windows. When the full XUV spectrum is considered, the transform-limited duration is approximately 500 as (solid black curve in Supplementary Fig. 6(a)), whereas restricting the analysis to the upper spectral portion increases the transform-limited duration to about 1 fs (dashed red curve in Supplementary Fig. 6(a)). Despite this reduction in bandwidth, the resulting broadening of the energy-integrated SB+ trace, and thus of the retrieved IRF, is only about 200 as. Therefore, the temporal resolution inferred from the experimentally

accessible part of the SB+ signal remains a reliable estimate of the overall instrument response. It's worth noting that, regardless of the portion of XUV spectrum considered, the IRF is largely dominated by the UV pulse profile (dash-dotted purple curve in Supplementary Fig.6(c)).

Another interesting characteristics of the experimental spectrogram of Fig. 2(d) of the main manuscript is the tilt of the SB+ feature, which is indicative of chirp in the XUV or UV pulses. In general, the tilt of a sideband spectrogram is determined by the chirp of both pulses, and a simultaneous measurement of SB+ and SB− is required to disentangle their respective contributions [4]. In the present case, however, there are strong reasons to conclude that the observed tilt arises predominantly from the UV pulse. First, an independent characterization of the UV generation sets its duration to $\Delta t_{UV} = 3.1$ fs. If one applies the standard expression for the cross-correlation of Gaussian pulses, $\Delta t_{SB+} = \sqrt{\Delta t_{UV}^2 + \Delta t_{XUV}^2}$ the experimentally observed sideband duration, $\Delta t_{SB+} = 3.2$ fs, is reproduced by assuming an effective XUV pulse duration of 800 as. This value is fully consistent with the transform-limited duration expected for the portion of the XUV spectrum that contributes to the upper part of the SB+ signal. Second, the transform-limited duration of the UV pulse is $\Delta t_{UV} = 2.1$ fs. If one were to attribute the observed cross-correlation width of 3.2 fs to an unchirped UV pulse, the same formula would imply an effective XUV pulse duration of $\Delta t_{XUV} = 2.41$ fs, corresponding to a GDD of the order of 1 fs^2. In our experimental configuration, however, no plausible physical mechanism is expected to induce such a large chirp in the selected portion of the XUV spectrum. The attochirp is typically on the order of 10^{-2} fs^2, while femtochirp becomes relevant only when high harmonics are generated with a long IR driving pulse, which is not the case in our setup [5]. These considerations justify our choice to perform the calculations reported in this section by assuming transform-limited attosecond XUV pulses. Including the residual attochirp of the XUV radiation and the atomic delay introduced by the argon target would produce only a minor correction to the estimated IRF and would not alter the conclusions of the analysis.

3 trPES simulations

3.1 Spectroscopy simulation

The simulation of the transient signals was performed on top of the surface hopping dynamics simulations from reference [6]. There, 103 trajectories were considered, initiated from geometries corresponding to excitations in the 260-280 nm range. To account for the broader spectral bandwidth of the pump pulse employed in the present experiment, we extended the original set of trajectories by including an additional ensemble of ∼30 surface hopping simulations initiated from geometries corresponding to excitations in the 280–305 nm range, which was not covered in Ref. [6]. This extension reflects the slightly larger portion of the vibronic phase space accessible under the present excitation conditions, as inferred from the analysis of the Wigner-sampled initial conditions. The inclusion of these additional trajectories does not lead to appreciable changes in the simulated observables, confirming that the original trajectory pool already captures the dominant features of the dynamics (see Supplementary

Fig. 7). For the simulation of trPES spectra we have employed the pump-probe simulation utility available with COBRAMM [7, 8]: we have calculated the energies of the lowest 6 ionized states (XMS-CASPT2/SA6-CASSCF(9,8)/cc-pVDZ) at intervals of 1 fs along each surface hopping trajectory, together with the Dyson intensities of the transitions associated with the active state at each time. In this way, the electron binding energy is sampled across a wide range of geometries, accounting for the modulation of the signal that could arise from differences in the topologies of potential energy surfaces of the valence/ionized states. This data was convoluted in energy and time to obtain the simulated trPES spectrum of acetylacetone in the gas phase (broadening parameters: time FWHM =3 fs, energy FWHM = 0.59 eV).

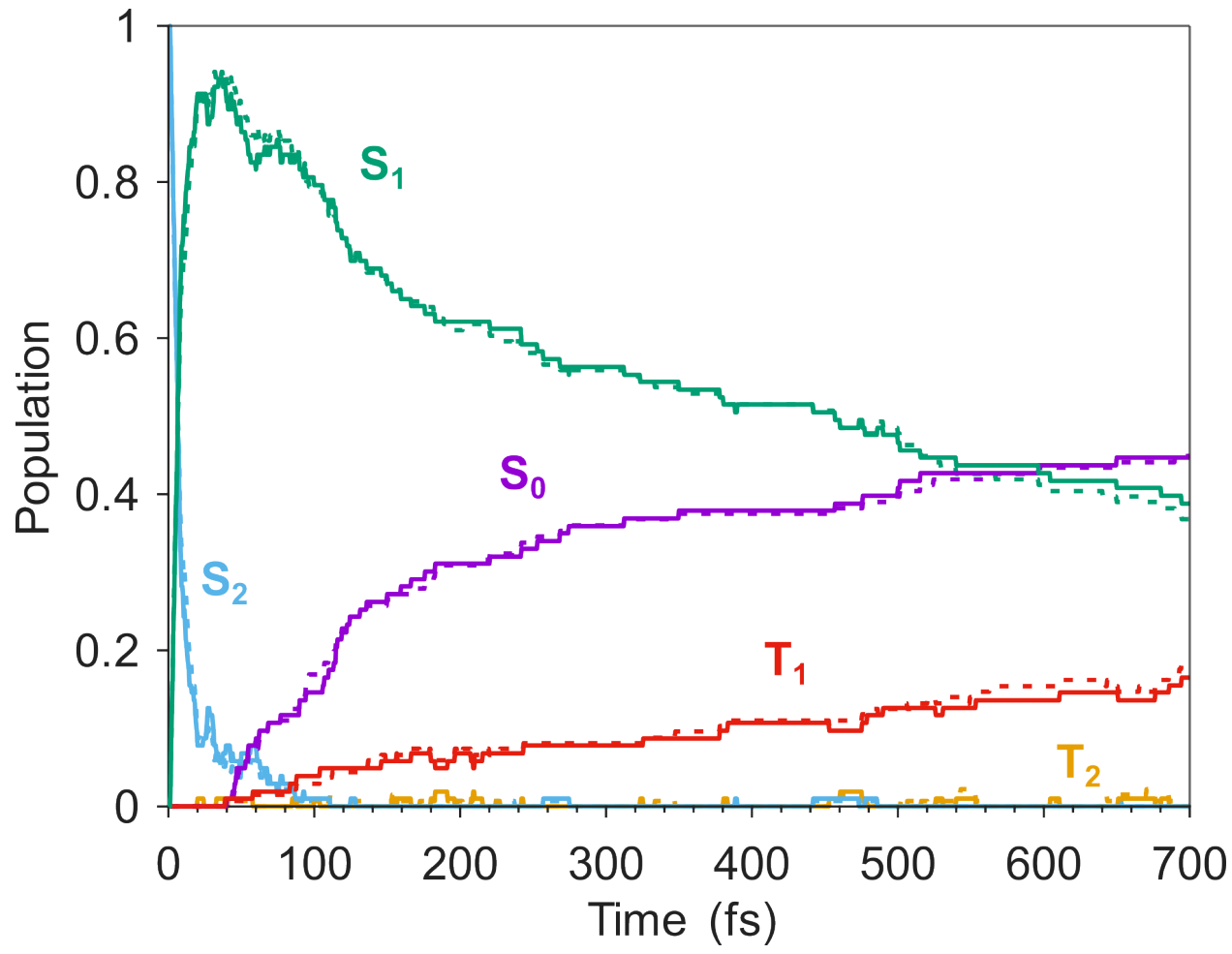

Supplementary Fig. 7 | Population dynamics comparison. Comparison of the population dynamics using the 103 trajectory-ensemble from reference [6] (full lines) and the 136 trajectory-ensemble that accounts for the broader excitation pulse of the present experiment (dashed lines). S_2 and S_1 denote the singlet excited states discussed in the main text, T_2 and T_1 the triplet states, and S_0 the ground state.

3.2 Decomposition of simulated trPES contributions

Simulations allow to disentangle the global trPES signal into contributions from different electronic states and/or contributions from various groups of trajectories showing similar behavior, providing further interesting insight into the origin of the various spectroscopic features and their connection with molecular motion on the different electronic state potential energy surfaces.

We have performed two distinct analysis, in order to investigate the connection of (i) the electronic state character and (ii) the displacement along ESIHT coordinate with the observed spectroscopic features. Concerning the former, we have chosen to

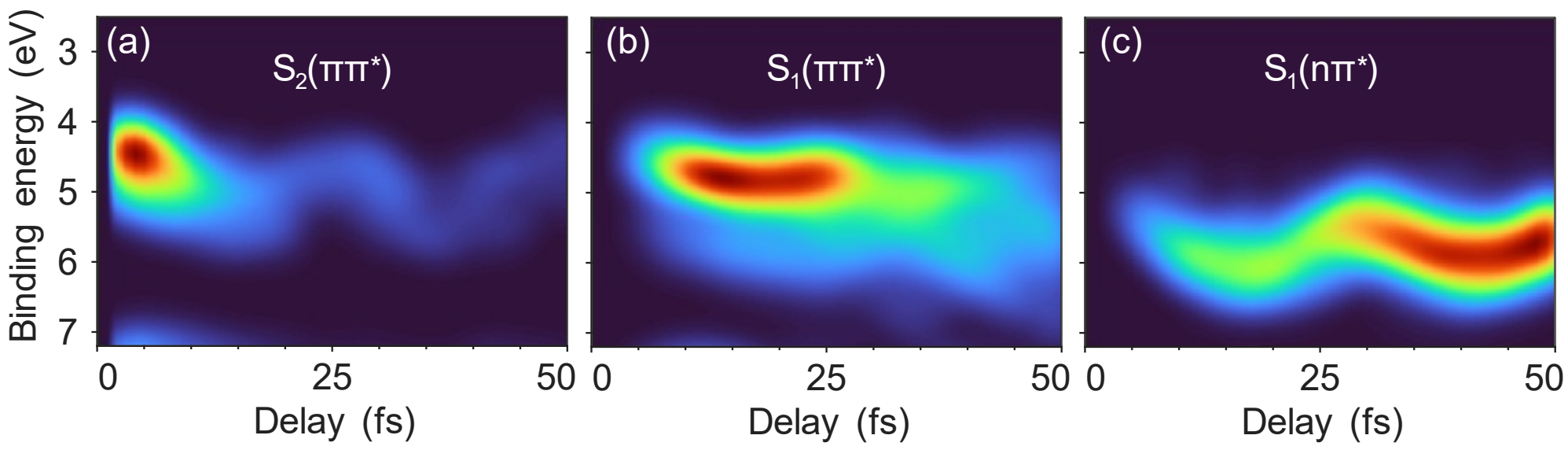

Supplementary Fig. 8 | Decomposition of simulated trPES. Contributions to the simulated trPES signals from (a) S_2, (b) S_1, when it shows a dominant $\pi\pi^*$ character and (c) S_1, when it shows a dominant $n\pi^*$ character (see paragraph 3.2 for definition of electronic state character).

assign the electronic character of the active state (for each trajectory and at each time) using the following criterium:

$$\pi\pi^* \quad \text{if} \quad |c_{\pi\pi^*}|^2 \geq 0.5$$
$$n\pi^* \quad \text{if} \quad |c_{n\pi^*}|^2 \geq 0.5$$

where c_i is the coefficient of electronic configuration i in the XMS-CASPT2 wavefunction.

S_2 is always showing $\pi\pi^*$ character, and its contribution to the trPES signal is shown in Supplementary Fig. 8(a). The separate contributions from S_1 when it shows dominant $\pi\pi^*$ or $n\pi^*$ character are shown in Supplementary Fig. 8(b) and (c), respectively. We note that, at each time step, the small but non-negligible portion of trajectories for which S_1 was showing a mixed $\pi\pi^*/n\pi^*$ character (i.e., $|c_{\pi\pi^*}|^2 < 0.5$ and $|c_{n\pi^*}|^2 < 0.5$) were excluded from this analysis.

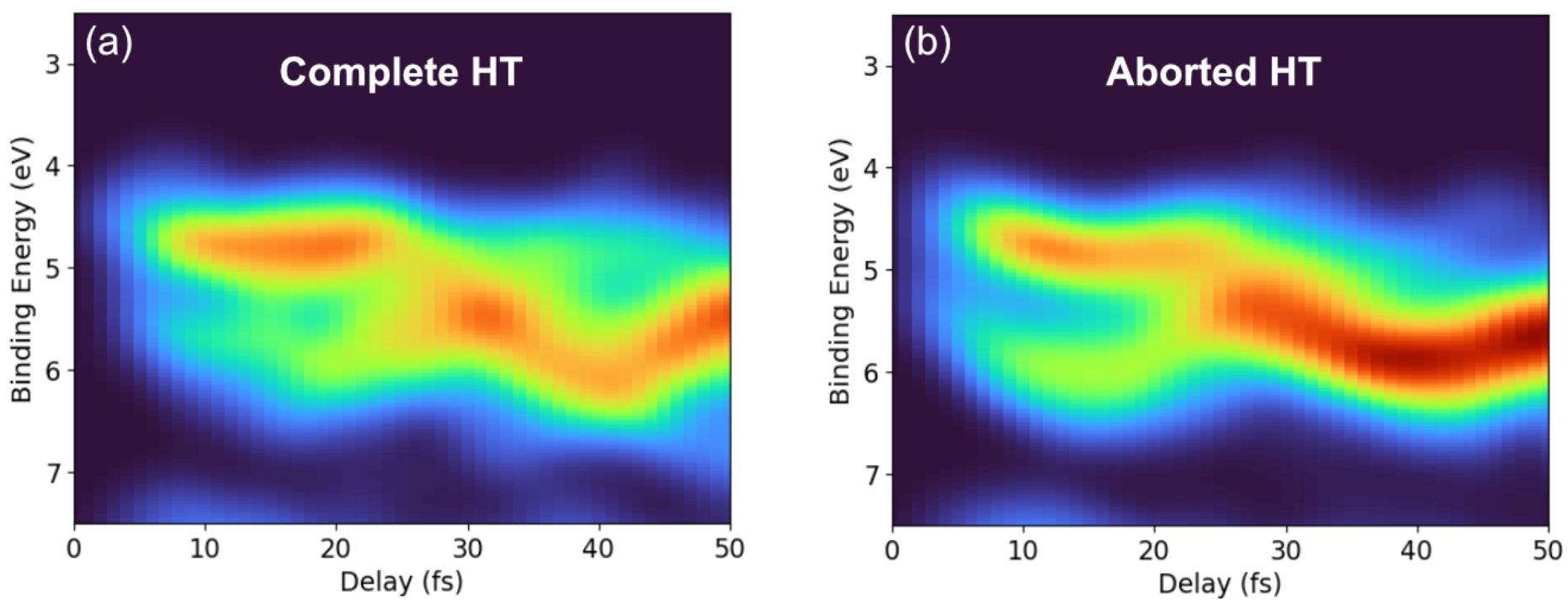

Supplementary Fig. 9 | trPES and hydrogen transfer. Contributions to the simulated trPES signals from (a) trajectories that complete hydrogen transfer, (b) trajectories for which the hydrogen transfer is aborted after some oscillations (ratio complete HT : aborted HT is 33:67 [6]).

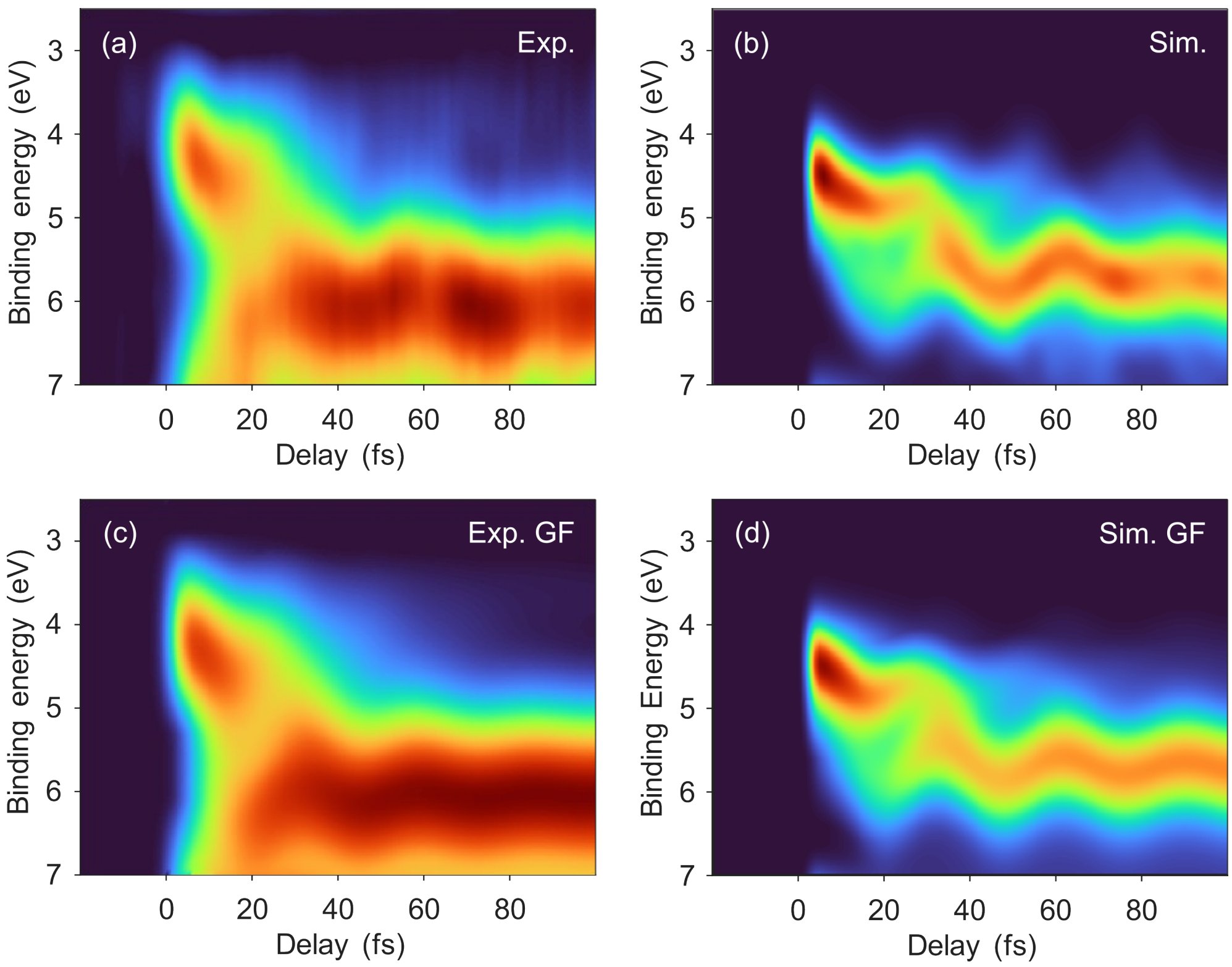

Supplementary Fig. 10 | Global fit analysis. Experimental (a) and simulated (b) trPES map. Global fit of the experimental (c) and simulated (d) data.

Concerning the assessment of the effect of the ESIHT on the observed signals, we have separated the surface hopping trajectories in two groups, based on the values of the two O-H distances at the beginning and end of the simulation. The signal originated by those trajectories that complete the $\mathrm{O-H\cdots O} \longrightarrow \mathrm{O\cdots H-O}$ transfer is shown in Supplementary Fig. 9(a), while the signals originated by trajectories in which the ESIHT is aborted is shown in Supplementary Fig. 9(b). The surface hopping trajectories are those from reference [6], and ESIHT is aborted in 67% of the cases.

i	γ (fs)	τ_r (fs)	τ_d (fs)	T_{osc} (fs)	τ_{damp} (fs)
1	3		8.8 ± 0.3		
2	3	8.8 ± 0.3	15.4 ± 0.4	28.1 ± 0.1	
3	3	15.4 ± 0.4		28.1 ± 0.1	35.5 ± 1.7

Supplementary Table 2 | Parameters retrieved from the global fit analysis of the experimental data, for the three spectral components ($i = 1, 2, 3$) described in the text.

i	γ (fs)	τ_r (fs)	τ_d (fs)	T_{osc} (fs)	τ_{damp} (fs)
1			8.1		
2		8.1	17.2	25	
3		17.2		27	35

Supplementary Table 3 | Parameters retrieved from the global fit analysis of the simulated data, for the three spectral components ($i = 1, 2, 3$) described in the text.

3.3 Discrepancies between experimental and calculated trPES traces

The simulated spectral features are narrower than the experimental ones by design, in order to better resolve the vibronic structure underlying the photoelectron signal. At the same time, the first band in the simulations (S_2 signal) is slightly blue-shifted compared to the experiment. This originates from the procedure used to account for the finite pump bandwidth: the simulated signal is averaged (via convolution) over the first 3 fs of dynamics to mimic the associated spectral uncertainty. While this provides a practical approximation, it introduces an artificial blue-shift of the initial signal ($t = 0$) with respect to the Franck–Condon (FC) energy. Indeed, the FC vertical ionization energy itself (4.16 eV) is in much better agreement with the experimental binding energy of the first peak. We also note that this approximate treatment of the finite pulse duration likely contributes to the overestimation of the intensity of the first signal relative to the experiment.

4 Static Photoelectron Spectrum

The static XUV photoelectron spectrum of acetylacetone is shown in Supplementary Fig. 11.

The calculated static spectrum displays four main ionization channels from the ground state (S_0), located at 8.92 eV ($S_0 \rightarrow D_0$), 9.47 eV ($S_0 \rightarrow D_1$), 12.19 eV ($S_0 \rightarrow D_2$), and 13.25 eV ($S_0 \rightarrow D_3$). The electronic structure analysis shows that each ionic state is dominated by a single leading configuration (corresponding to ionizations from π HOMO, n HOMO-1, π HOMO-2 and π HOMO-3 orbitals, respectively), indicating a largely single-hole character with moderate configuration mixing. In view of the relatively broad bandwidth of the XUV radiation exploited in the experiment, the experimental static spectrum displays only two main features, corresponding to the first pair ($S_0 \rightarrow D_0$ and $S_0 \rightarrow D_1$) and the second pair ($S_0 \rightarrow D_2$ and $S_0 \rightarrow D_3$) of ionization channels, respectively.

5 Global fit reconstructions

To isolate the independent components that define the trPES spectrum shown in Fig. 3 of the main text, we apply a least-squares fitting model to the trPES map, PES(E, τ),

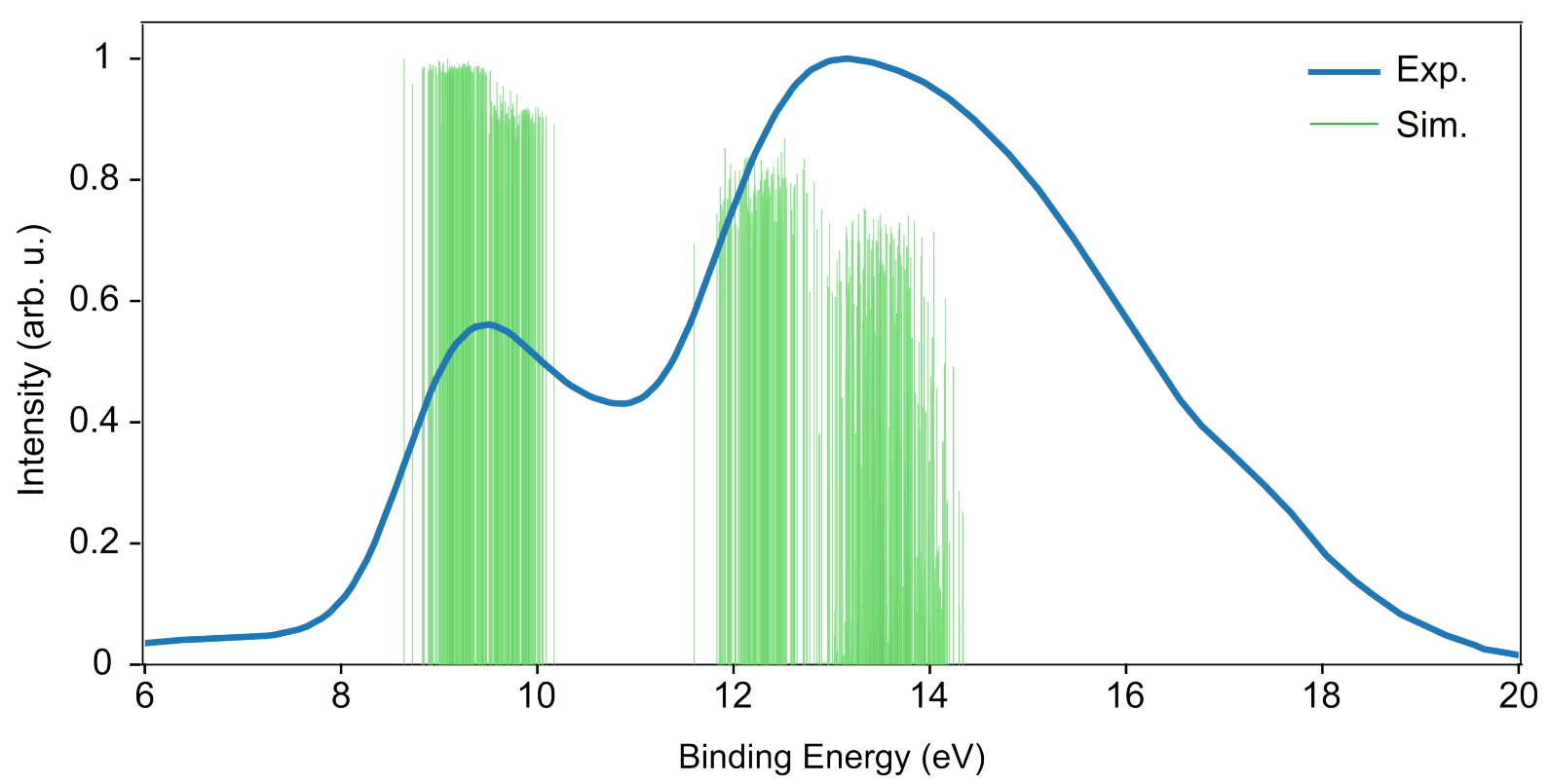

Supplementary Fig. 11 | Static XUV photoelectron spectrum of acetylacetone. The experimental spectrum is shown in blue, while the green sticks represent the simulated spectral contributions.

where E denotes the photon energy and τ the pump-probe delay, as follows:

$$\mathrm{PES}(E,\tau) = \sum_{i=1}^{3} A_i(E) f_i(\tau) \qquad i = 1,2,3 \tag{1}$$

where $A_i(E)$ are energy-dependent amplitudes and $f_i(\tau)$ are temporal response functions.

All temporal components are modeled as the convolution of a common Gaussian instrument response function (IRF) with underlying kinetic populations $F_i(\tau)$:

$$f_i(\tau) = \big[\mathrm{IRF} * F_i\big](\tau), \tag{2}$$

where the IRF is written as

$$\mathrm{IRF}(\tau) = \frac{1}{\gamma\sqrt{2\pi}} \exp\Big(-\frac{\tau^2}{\sqrt{2}\gamma^2}\Big). \tag{3}$$

The underlying populations $F_i(\tau)$ follow a sequential first-order kinetic scheme $F_1 \xrightarrow{k_1} F_2 \xrightarrow{k_2} F_3 \xrightarrow{k_3}$ sink, with $k_i = 1/\tau_{d_i}$. Using the Heaviside step function $H(x)$ to enforce causality, the populations are

$$F_1(\tau) = H(\tau)\,\exp\left[-\frac{\tau}{\tau_{d_1}}\right], \tag{4}$$

$$F_2(\tau) = H(\tau)\,\frac{k_1}{k_2 - k_1}\,\left[e^{-k_1\tau} - e^{-k_2\tau}\right], \tag{5}$$

$$F_3(\tau) = H(\tau)\,\left[c_1 e^{-k_1\tau} + c_2 e^{-k_2\tau} + c_3 e^{-k_3\tau}\right], \tag{6}$$

with

$$c_1 = \frac{k_1 k_2}{(k_2 - k_1)(k_3 - k_1)}, \tag{7}$$

$$c_2 = \frac{k_1 k_2}{(k_1 - k_2)(k_3 - k_2)}, \tag{8}$$

$$c_3 = \frac{k_1 k_2}{(k_1 - k_3)(k_2 - k_3)}. \tag{9}$$

In this picture, the first component $A_1(E)f_1(\tau)$ reproduces the signal centered around ~ 4 eV, whose rise is defined by the pump-induced electronic excitation and which decays with time constant τ_{d_1}. The second and third components, $A_2(E)f_2(\tau)$ and $A_3(E)f_3(\tau)$, describe the population sequentially transferred to the bands centered at ~ 5 eV and ~ 6 eV with decay times τ_{d_2} and τ_{d_3}, respectively.

The global-fit was performed using an oscillatory model that explicitly accounts for the center of mass modulations of the individual spectral components. This is implemented allowing the spectral components $A_i(E)$ to oscillate in energy with a sinusoidal function, of which the fit retrieves period (τ_{osc}), phase and damping time (τ_{damp}). The experimental fitting results and their errors are obtained, respectively, as mean and standard deviation of the results of the global fit analysis performed on each of the measured scans. Supplementary Fig. 10 shows the comparison between the data and the result of the fitting procedure, both for the experimental and simulated data. The retrieved spectral ($A_i(E)$) and temporal ($f_i(\tau)$) components are shown in Fig. 3 of the main text. Tables 2 and 3 present the corresponding kinetic parameters retrieved from the global fit analysis.

Supplementary References

[1] Madsen, L.B.: Strong-field approximation in laser-assisted dynamics. American journal of physics **73**(1), 57–62 (2005)

[2] Reduzzi, M., Pini, M., Mai, L., Cappenberg, F., Colaizzi, L., Vismarra, F., Crego, A., Lucchini, M., Brahms, C., Travers, J.C., Borrego-Varillas, R., Nisoli, M.: Direct temporal characterization of sub-3-fs deep uv pulses generated by resonant dispersive wave emission. Opt. Express **31**(16), 26854–26864 (2023)

[3] Lucchini, M.: Floquet interpretation of dynamic interference in laser-assisted photoemission. Physical Review A **112**(4), 043120 (2025)

[4] Moio, B., Dolso, G.L., Inzani, G., Di Palo, N., Borrego-Varillas, R., Nisoli, M., Lucchini, M.: Time-frequency mapping of two-colour photoemission driven by harmonic radiation. Journal of Physics B: Atomic, Molecular and Optical Physics **54**(15), 154003 (2021)

[5] Isinger, M., Busto, D., Mikaelsson, S., Zhong, S., Guo, C., Salières, P., Arnold, C., l'Huillier, A., Gisselbrecht, M.: Accuracy and precision of the rabbit technique.

Philosophical Transactions of the Royal Society A: Mathematical, Physical and Engineering Sciences **377**(2145) (2019)

[6] Severino, S., Aleotti, F., Mai, L., Crego, A., Medeghini, F., Frassetto, F., Poletto, L., Lucchini, M., Segatta, F., Reduzzi, M., Nisoli, M., Nenov, A., Borrego-Varillas, R.: Mapping excited-state decay mechanisms in acetylacetone by sub-20 fs time-resolved photoelectron spectroscopy. Journal of the American Chemical Society **147**, 30785–30793 (2025)

[7] Avagliano, D., Bonfanti, M., Nenov, A., Garavelli, M.: Automatized protocol and interface to simulate QM/MM time-resolved transient absorption at TD-DFT level with COBRAMM. Journal of Computational Chemistry **43**(24), 1641–1655 (2022)

[8] Cobramm Wiki. https://gitlab.com/cobrammgroup/cobramm/-/wikis/home. Accessed on 2025-08-02 (2025)
