# Supplementary material for: 3-fs-resolved UV/XUV photoelectron spectroscopy in the gas phase
Source: Nat Commun. 2026 Aug 19;17:9912. doi: 10.1038/s41467-026-76602-4 (PMC13582955; doi:10.1038/s41467-026-76602-4)
Supplement: Supplementary file 2 [file 41467_2026_76602_MOESM2_ESM.pdf]

nature portfolio

Peer Review File

3-fs-resolved UV/XUV photoelectron spectroscopy in the gas phase

Corresponding Author: Professor Maurizio Reduzzi

This file contains all reviewer reports in order by version, followed by all author rebuttals in order by version.

Version 0:

Reviewer comments:

Reviewer #1

(Remarks to the Author)
Key Results:

Reduzzi et. Al have reported a new experimental setup which utilizes a combination of Sn filtered high harmonic driven extreme ultraviolet (XUV) source and a resonant dispersive wave source generating few cycle ultraviolet pulses for experiments studying the dynamics of molecules with few (~3 fs) femtosecond temporal resolution. They demonstrate the temporal resolution of the setup utilising a cross-correlation measurement in noble gases. The bulk of the paper is dedicated to describing results using this setup as applied to the prototypical acetylacetone molecule, whereby the UV pulse is used to drive a transition to the $\pi\pi*$ state, which then rapidly relaxes through a CI to the $n\pi*$ state. In comparing the results from their setup to those of previous measurements with a longer instrument response (20 fs) by Severino et. al. they identify additional fast dynamical components which were not resolvable in the latter. The experimental results are compared to CASPT2, and a global fit analysis is applied to be both the theoretical and experimental results, which show good agreement.

Suitability of Publication:

In the reviewer's opinion, the overall quality of the paper and the supplementary material is more than satisfactory to warrant publication in some form. The methodology, theoretical and experimental details are described in sufficient detail that they could be independently replicated by other research groups, and both the theoretical and experimental results are communicated clearly and thoroughly. This reviewer found no flaws in the presented data or methodology that would justify outright refusal of publication.

UV-XUV setups and RDW UV pulse generation have been previously established in literature, so to evaluate whether the paper's significance is sufficient to warrant publication in Nat. Comm. specifically, one must consider whether whether the combination of the improved UV pulse duration via RDW combined with HHG in a pump probe setup, the claimed improvement in temporal resolution compared to other setups, and the prototypical AcAc results included in the paper demonstrate a truly "significant advancement to specialists in the field", as defined by Nat. Comm's mission statement.

To the reviewers knowledge, the only paper that demonstrates similar temporal resolution as concerns the UV pulse duration in combination with HHG XUV pump-probe capability in a tabletop laboratory setup is is the setup by V. Wanie et. al, (partially described by citation [5], more completely in Rev. Sci. Instrum. 95, 083004 (2024)) which instead uses third harmonic generation for the UV generation. In their setup they also demonstrate UV pump - XUV probe data, however this is has been limited to ion data, and to my knowledge they have not yet published data demonstrating any trPES measurement capability.

By this reviewers' reckoning, the unique properties of the setup described in this paper compared to those described in literature are the following:

a) Tunability of the UV spectrum over a broad range with reasonable pulse energy. Compared to THG, which is locked to the drive laser's tunability and limited to approximately 100 uJ, the authors are able to demonstrate being able to deliver in excess of 500 uJ over 100 nm of UV tuning range, which will be advantageous in being able to target specific transitions in a

broad range of physically and biologically interesting molecules.

b) Their data is obtained utilizing relatively narrowband HHG pulse(s?) which offers significantly improved resolution of the different electronic states of the target molecule compared schemes utilizing broadband harmonic spectra, while maintaining an overall temporal resolution of only a few femtoseconds.

c) Their data does illuminate dynamics on timescales that are significantly faster than past "pure" pump-probe measurements, and the scheme they propose broaches the gap between traditional femtosecond pump-probe schemes and attosecond interferometry and IAP pump/probe schemes in both the timescales it can probe and the degree by which the electronic states can be resolved, at least as concerns tabletop driven sources.

I feel these factors are demonstrative of an advance significant enough to fully justify publication in a journal such as Nat. Comm.

There are a few questions I would like addressed before I can recommend this paper for publication unconditionally. None are deal-breakers, but I think could potentially improve the manuscript if they are addressed and would provide additional valuable information to a reader in the field.

- Can the authors elaborate on what they expect the temporal structure of the XUV to be under their experimental conditions? In the manuscript they describe the high harmonic source being able to produce photon energies between 20-40 eV and can support isolated pulses of duration <= 180 as. However, given they are filtering only relatively low energy harmonics and are (I presume) far from the continuum cut-off region normally used for IAP generation (from the spectrum it looks like the authors have 2 near-discrete harmonics) then I would assume you would have some kind of pulse train, or at least some satellites? The reviewer was able to find a Master's Thesis by M. Trevisan which appears to describe the same/similar setup, where the transform limited duration was calculated to be 450 as, albeit with satellites about 15% of the main pulse height. I think including a brief explanation of this (or at least an upper limit for the XUV pulse duration similar to those stated for the UV pulses) in either the text or supplementary material could be useful in establishing what the current limits are for their temporal resolution (and potential for improvement in future!).

- Related to this: could the authors comment briefly on the reasons for the asymmetric shape/slope of the 2D sideband cross correlation trace?

- If I am not mistaken, the ETF15 the authors utilize for electron spectroscopy can also be used in a positive ion mode. Given the challenge with signal/noise in the sideband measurements, I wonder if the authors considered/tried using ion yield measurements as an alternative/independent cross correlation scheme? I believe their total XUV+UV energies might be sufficient to conditionally ionise helium only in a XUV+UV two photon scheme, for example, and the mass selectivity could make the measurements less sensitive to background compared to electron detection.

Other than these two questions I have only a few minor technical Points and suggestions for improvement:

- I think "Ultraviolet photoinduced dynamics" in the introductory sentence would read better as "Ultraviolet-photon induced electron dynamics" or "Electron dynamics induced by photoabsorption in the ultraviolet".
- In figure 1, FM (focusing mirror) and hollow core fibre (HCF) are not defined in the figure caption. While this is explained in text I think its always better if the captions are independently intelligible.
- In figure 2 (a), I think it would be useful to indicate the expected electron kinetic energies of the MB and SB peaks in some way, or at least make the distinction between bound states and continuum electrons in some way (maybe by shading the continnum or bound states.
- Figures 2 and 3 look a little congested as currently laid out. While I understand space is sometimes at a premium, I think a little more white space between some of the elements would go a long way to make the different elements more distinct from each other and improve the readability of the figures.
- In the lower part of Fig. S1 it seems like the colour of data points in the pulse energy graphs don't have a 1:1 correspondence to the colours in the scales in the spectral plots above them. I think these subplots would benefit from either having independent colour scales or utilising some other way to indicate the pressure for each point. As it stands, it's non-trivial to gain anything too quantitative from the plot colours.

Conclusion:

In summary, if the above are satisfactorily addressed through minor additions/changes, I would recommend that the editors approve the publication of this article.

Reviewer #2

(Remarks to the Author)
The manuscript entitled “3-fs resolved UV/XUV photoelectron spectroscopy in the gas phase” by M. Pini and coworkers reports on a (to the best of my knowledge) first time-resolved photoelectron spectroscopy experiment employing an ultrashort UV pump pulse from resonant dispersive wave (RDW) emission and an isolated attosecond XUV probe pulse. The beamline is presented and the time resolution is characterized, and then a proof-of-principle experiment in a prototypical molecule undergoing ultrafast internal conversion, acetylacetone (AcAc), is performed. The time-resolved photoelectron spectra are analyzed with a global fitting method to extract the relevant time constants. The experimental data are compared

to simulated time-resolved photoelectron spectra where the dynamics are calculated with trajectory surface hopping and the photoelectron spectra are determined taking into account six cationic states. Experimental and simulated time-resolved photoelectron spectra appear in very good agreement. The authors had performed a similar experiment in AcAc with a 20-fs UV pump pulse (Ref. 38), and the use of a much shorter (and therefore spectrally broader) UV pump gives rise to transient features not observed in Ref. 38.

This work combines two state-of-the-art ultrashort pulses techniques (RDW + isolated as XUV) in an extremely challenging experiment. Ultrashort UV pulses from RDW is an emerging technology with a transformative potential for ultrafast sciences, because it gives access to few-fs broadband and tunable pulses in the UV. It's just beginning to be used in time-resolved experiments, with only a few reported works (Refs. 2, 15, 16, 17) and none of them using isolated attosecond XUV pulses. The results reported by Pini et al. in the present manuscript are therefore highly significant for ultrafast dynamics as it demonstrates time-resolved photoelectron spectroscopy with RDW UV and isolated attosecond XUV pulses, with an instrument response function of the order of just a few fs. The quality of the data is very good and the paper is well written. I think the manuscript can deserve publication in Nature Communications, after the questions raised by the use of ultrashort and therefore spectrally broad spectra for both the pump and the ionizing pulses are addressed in more detail.

Indeed, the few-fs UV pump pulse used for excitation shown in Fig. 2b spans 260-305 nm fwhm, which is over 700 meV fwhm bandwidth. As written by the authors in the manuscript, such a pump pulse can coherently excite multiple electronic (and a fortiori vibrational) states in a system, and I think that this point (very important for the future use of RDW UV pump in ultrafast spectroscopy and even "attochemistry"!) is not sufficiently discussed in the manuscript. Secondly, the probe pulse used for ionization displayed in Fig.2c spans over more than 4 eV. One can imagine that the ionization of a molecule with such a pulse leads to large, overlapping photoelectron bands. In the time-resolved photoelectron spectra presented in Fig. 3e, the authors seem to be able to identify features that are spectrally narrow compared to the ionizing probe pulse. To address these points, I would like the authors to:
1) Justify the use of the same trajectories as in Ref. 38 in the simulation. Ref. 38 used a pump pulse with just 250 meV bandwidth fwhm compared to over 700 meV here. The trajectories from Ref. 38 were selected based on the spectrum of the excitation pulse of Ref. 38 which is different from the much broader spectrum used here. Can the broad pump spectrum coherently excite vibronic dynamics in AcAc?

2) Along the same lines, how are the timescales and branching ratios discussed on p.10 and shown in Fig.4 dependent on the initial excited state population (and therefore on the spectral bandwidth of the pump pulse)? The authors write multiple times in the manuscript that their experiment in AcAc enables the observation of dynamics that have remained "unresolved" or "invisible" due to a lack of temporal resolution… But is it really the same dynamics if a narrow or broad pump pulse is used to trigger them?

3) The authors should show and comment the static XUV only photoelectron spectrum of the molecule. From the data in Fig. 3e, it looks like the part of the XUV spectrum peaked around 22 eV is mainly contributing to the ionization. However, a discussion on the ability to get "good" spectral resolution in the photoelectron spectra despite using a 4-eV broad ionizing pulse is needed.

4) Along the same lines, in the dressed photoelectron spectrum used for the characterization of the instrument response function shown in Fig. 2d, the width of SB+ shown is much smaller than the width of the XUV pulse. Because of the large XUV spectrum, some of SB+ probably overlaps with the MB that cannot be measured for technical reasons. Therefore, contrary to Refs. 32 and 33 which used more "monochromatic" XUV, it seems that not all of SB+ signal is integrated to determine the IRF. The IRF is thus probably longer than the 3.2 fs given here? In addition, the SB+ appears tilted in Fig. 2d – is this the sign of a chirp in either the XUV or UV pulses (or both)?

5) The n-pi* and pi-pi* states should be distinguishable in the photoelectron spectrum because they would primarily ionize to different states of the cation (see e.g. DOI 10.1021/jacs.4c02886). This is suggested by the decompositions of the simulated tr-PES in Fig.S4 b and c, but a more explicit discussion of this point would be valuable in the context of tr-PES with XUV pulses where multiple cationic states are accessible.

In addition, I have a few minor comments:
6) What do you mean by "full and aborted H transfer" on p. 10? Do you refer to the "33%" path in Fig. 4 as full H transfer and the other paths in Fig. 4 as aborted H transfers?

7) The RDW spectra in Fig. 1b seem to correspond to the Argon spectra in Fig. S1, but the legend of Fig.1 in the main text reads "neon" and so does the text on p.5. Please correct the gas used.

8) The methods section only gives the maximum estimated UV peak intensity. For reproducibility purposes, please add the estimated peak intensities in the experiments reported in Fig. 2d and 3e.

9) When discussing the molecular structure of AcAc (shown in Fig. 3a), a precision that the enolic form is dominant in the gas phase would benefit a non-specialist community.

10) The colors used in Fig. 4 are hardly distinguishable if the figure is printed in shades of grey.

Reviewer #3

(Remarks to the Author)
The manuscript by Pini et al. describes a new UV–XUV pump–probe beamline for time-resolved photoelectron spectroscopy (trPES) combining ultrashort UV pump pulses with attosecond XUV probe pulses to reach an impressive temporal resolution. Using UV–XUV sideband cross-correlation in a noble gas, the authors report an in situ instrument response function of 3.2 ± 0.2 fs. Because the XUV probe is only a few hundred attoseconds long, the overall temporal resolution is largely set by the UV pump duration, which can be as short as a few femtoseconds when combining UV generation using the resonant dispersive wave (RDW) technique with careful dispersion management, as the authors have also shown in their previous work.

As a proof-of-principle application, the authors study the UV-induced dynamics of acetylacetone. They use a global-fit model to decompose the trPES data into three components and, supported by CASPT2-based simulated trPES, interpret the dynamics as two sequential passages through the S_2/S_1 conical intersection (CI). They conclude that the apparatus enables direct, time-domain observation of coupled electronic–nuclear wave-packet evolution in increasingly complex molecular systems.
Overall, the manuscript is well written, the technical execution impressive, and the demonstrated timing capability will be broadly useful for ultrafast laboratories pursuing studies of sub–10 fs chemical dynamics. That said, there are several important questions regarding the acetylacetone study that need to be clarified in order for the manuscript to be suitable for publication in Nature Communications.

Major questions
1. Broadband excitation vs. single-state (“prepared S_2”) narrative: The manuscript emphasizes the broad bandwidth of both the UV pump and XUV probe pulses and frames this capability as enabling precise excitation/probing of coherent superpositions central to “attochemistry.” However, the mechanistic interpretation for acetylacetone appears to be closely aligned with earlier work—primarily a refinement in temporal resolution rather than a qualitatively new dynamical picture. Given that the bandwidth of the UV pulse spans multiple eV, the authors should (i) justify why the initially prepared wave packet is dominated by a single electronic state (S_2) rather than a mixture of different excited states accessible within the photon energy range covered by the excitation pulse, and (ii) discuss why signatures of electronic coherence are not apparent in the observables.

2. Role of triplet states and early-time ISC: The mechanistic model and simulations appear to neglect triplet pathways. While singlet -> triplet intersystem crossing (ISC) is often reported on ~1.5 ps timescales, the earlier work used for comparison (Severino et al., J. Am. Chem. Soc. 2025, 147, 30785–30793) suggests some population transfer to T_1 within the first ~100 fs. Can the authors clarify the rationale for excluding the triplet states here and state explicitly whether this experiment is sensitive to early ISC (e.g., via distinct photoelectron signatures), whether any such contribution is unresolved within the fit, or whether it is spectroscopically overlapped with singlet features?

3. Experiment–theory mismatches: Experiment and simulations show very good agreement in terms of the timescales of the extracted components (Fig. 3(g)). However, the readers would benefit from a more explicit discussion of where the simulated trPES deviates from the experiment (e.g., binding-energy positions, relative intensities in Fig. 3(f,i)) and why this is the case.

Minor comments
1. The simulated spectra are convolved with a temporal FWHM of 3 fs and an energy FWHM of 0.59 eV. Please justify the 0.59 eV broadening chosen for the effective experimental energy resolution.

2. The manuscript argues that many trPES experiments are limited to ~10 fs resolution, impeding access to sub–10 fs dynamics. It would be informative to convolve the simulated trPES with 10 fs (in addition to 3 fs) to show which qualitative signatures survive and to clarify why acetylacetone is a particularly compelling system for showcasing the improvement.

3. The authors state that they “directly” resolve two CI passages. Improved time resolution can certainly reveal features that would otherwise be blurred, but the manuscript should define what is meant by “direct” in this context.

Reviewer #4

(Remarks to the Author)
I co-reviewed this manuscript with one of the reviewers who provided the listed reports. This is part of the Nature Communications initiative to facilitate training in peer review and to provide appropriate recognition for Early Career Researchers who co-review manuscripts.

Version 1:

Reviewer comments:

Reviewer #1

(Remarks to the Author)
This review is for NCOMMS-26-003532A, a revised version of the earlier reviewed paper titled "3-fs-resolved UV/XUV photoelectron spectroscopy in the gas phase"

It is in this reviewer's opinion that the authors have done a more than satisfactory job in addressing both my and the other three reviewer's comments and suggestions. The current iteration of the manuscript is a notable improvement over the first draft in terms of clarity and in the depth of key areas of the discussion/analysis. The in-depth description of the sideband measurement methodology in the supplementary material, and the addition of the discussion regarding Reviewer 2's excellent point regarding the coherent excitation of multiple states are both particularly welcome additions.

As it stands, I can wholeheartedly recommend publication.

Reviewer #2

(Remarks to the Author)
The authors have carefully addressed the points that I have raised. Through additional simulations, they show that the dynamics induced by the broad pump pulse are only due to electronic state S_2, like in their narrower pump work from Ref. 36 displayed in Fig. 3b. The addition of the static photoelectron spectrum of AcAc and of the SFA simulations of the dressed photoelectron spectrogram also allows a more thorough discussion of the probe pulse's spectral and temporal width effects on the measurements.
I have noticed a typo in the states characters at the bottom of revised article page 10 where it reads "where the S_2 (n pi*) and S_1 (pi pi*) surfaces…" instead of S_2 (pi pi*) and S_1 (n pi*).
I recommend the publication of the revised manuscript in Nature Communications.

Reviewer #3

(Remarks to the Author)
The authors have thoroughly revised the manuscript and have addressed all questions and comments raised by the reviewers in a satisfactory manner. I therefore recommend publication of the revised manuscript in Nature Communication.

Reviewer #4

(Remarks to the Author)
I co-reviewed this manuscript with one of the reviewers who provided the listed reports. This is part of the Nature Communications initiative to facilitate training in peer review and to provide appropriate recognition for Early Career Researchers who co-review manuscripts.

Open Access This Peer Review File is licensed under a Creative Commons Attribution 4.0 International License, which permits use, sharing, adaptation, distribution and reproduction in any medium or format, as long as you give appropriate credit to the original author(s) and the source, provide a link to the Creative Commons license, and indicate if changes were made.
In cases where reviewers are anonymous, credit should be given to 'Anonymous Referee' and the source.
The images or other third party material in this Peer Review File are included in the article's Creative Commons license, unless indicated otherwise in a credit line to the material. If material is not included in the article's Creative Commons license and your intended use is not permitted by statutory regulation or exceeds the permitted use, you will need to obtain permission directly from the copyright holder.

To view a copy of this license, visit https://creativecommons.org/licenses/by/4.0/

Manuscript #: NCOMMS-26-00353-T
Title: 3-fs-resolved UV/XUV photoelectron spectroscopy in the gas phase
Author: M.Pini *et al*.

List of changes:
In reply to the Reviewers' comments, we have revised the manuscript with the following changes.

Main text:

- The Introduction was expanded by adding a discussion of the role of the broad RDW UV pump bandwidth in coherently exciting multiple nearby electronic states and vibronic levels, together with its relevance for attochemistry-oriented applications.
- The caption for Figure 1 was revised to provide clear definitions for all symbols used.
- The spacing between panels in Figure 2 was increased. Panel 2(a) has been modified to mark a clearer distinction between bound and continuum states.
- The spacing between panels in Figure 3 was increased.
- In the instrumental-response-function section, we specified the estimated UV peak intensity used in the sideband measurement.
- We modified a few sentences of the section "UV pump – XUV probe in Acetylacetone", introducing the keto-enol tautomerism, specifying that we excite only one electronic state despite the broad pump bandwidth, and clarifying the different cationic states involved in the trPES trace.
- The simulations reported in Fig. 3 (h) (with the corresponding global fit analysis displayed in panels (i) and (j)) include an additional ensemble of about 20 surface-hopping trajectories covering excitations in the 280-305 nm range, in order to account for the broader pump bandwidth used in the present experiment.

Supplementary Information:

- We clarified the caption of Fig. S1 by specifying that panels (c) and (d) include a larger dataset than panels (a) and (b).
- We added Sect. 2 (Instrumental response function) to describe in detail how the IRF was evaluated through Strong-Field Approximation (SFA) calculations based on the experimental UV and XUV spectra. We added Figs. S4, S5 and S6.
- We largely revised Sect. 3.1 (Spectroscopy simulation). We expanded this section by including an additional ensemble of about 20 surface-hopping trajectories covering excitations in the 280-305 nm range, in order to account for the broader pump bandwidth used in the present experiment.
- We introduced Sect. 3.3 commenting on the remaining discrepancies between simulated and experimental trPES signals.
- We updated Table S3 with revised simulated global-fit parameters, including $\tau_{d,1} = 8.1$ fs, $\tau_{r,2} = 8.1$ fs, $\tau_{d,2} = 17.2$ fs, and $\tau_{r,3} = 17.2$ fs.
- We inserted a section, now Sect. 4 (Static Photoelectron Spectrum), to describe the XUV-only static photoelectron spectrum.

We are grateful to all Reviewers for their work and useful comments. We are confident that the new version of the manuscript fully addresses their concerns and meets the criteria for publication in Nature Communications.
In the following, we give our reply point by point.

We thank the Reviewers for the time and effort they devoted to reviewing our manuscript and for their valuable comments, which have significantly contributed to improving it. Below, we respond to each of their concerns point by point.

Reviewer #1 (Remarks to the Author)

Key Results:
…..
Suitability of Publication:
In the reviewer's opinion, the overall quality of the paper and the supplementary material is more than satisfactory to warrant publication in some form. The methodology, theoretical and experimental details are described in sufficient detail that they could be independently replicated by other research groups, and both the theoretical and experimental results are communicated clearly and thoroughly. This reviewer found no flaws in the presented data or methodology that would justify outright refusal of publication.

We thank the Reviewer for the positive comments and the careful reading of our manuscript.

UV-XUV setups and RDW UV pulse generation have been previously established in literature, so to evaluate whether the paper's significance is sufficient to warrant publication in Nat. Comm. specifically, one must consider whether whether the combination of the improved UV pulse duration via RDW combined with HHG in a pump probe setup, the claimed improvement in temporal resolution compared to other setups, and the prototypical AcAc results included in the paper demonstrate a truly "significant advancement to specialists in the field", as defined by Nat. Comm's mission statement.
To the reviewers knowledge, the only paper that demonstrates similar temporal resolution as concerns the UV pulse duration in combination with HHG XUV pump-probe capability in a tabletop laboratory setup is is the setup by V. Wanie et. al, (partially described by citation [5], more completely in Rev. Sci. Instrum. 95, 083004 (2024)) which instead uses third harmonic generation for the UV generation. In their setup they also demonstrate UV pump - XUV probe data, however this is has been limited to ion data, and to my knowledge they have not yet published data demonstrating any trPES measurement capability.

We thank the Reviewer for pointing out the work by V. Wanie *et al.*. We would like to clarify that one of the authors of the present manuscript (M. Nisoli) is also a co-author of the work mentioned by the Reviewer. We are therefore fully aware of that experimental implementation and of its scope.
That work is based on a different experimental strategy from the one presented here. In particular, the UV excitation is generated by third-harmonic generation, whereas in the present manuscript a completely different approach is used to produce few-femtosecond UV pulses. More importantly, the observables accessed in the two experiments are fundamentally different. The work by Wanie et al. reports ion yield as a function of pump-probe delay, whereas the present study is based on time-resolved photoelectron spectroscopy. For this reason, the cited work should not be considered as demonstrating the same type of ultrafast capability discussed in the present manuscript. In Wanie's paper, the UV-pump/XUV-probe measurements were mainly presented as a possible example of application of the developed beamline, rather than as a study aimed at resolving and discussing an ultrafast electronic dynamics. Accordingly, no specific ultrafast dynamics was highlighted there.
By contrast, in the present work, the use of UV pulses as short as about 3 fs enables us to resolve with high clarity the very first steps of the system evolution immediately following

excitation. This is precisely the key result and added value of the present study: not simply the implementation of a UV-pump/XUV-probe scheme in a tabletop setup, but the direct observation, through time-resolved photoelectron spectroscopy, of the earliest stages of the ultrafast dynamics.
We agree that the work by Wanie *et al.* is an important related contribution and we have now included it in the revised manuscript as Ref. [6].

By this reviewers' reckoning, the unique properties of the setup described in this paper compared to those described in literature are the following:

a) Tunability of the UV spectrum over a broad range with reasonable pulse energy. …
b) Their data is obtained utilizing relatively narrowband HHG pulse(s?) which offers significantly improved resolution …
c) Their data does illuminate dynamics on timescales that are significantly faster than …

I feel these factors are demonstrative of an advance significant enough to fully justify publication in a journal such as Nat. Comm.

We thank the Reviewer for the positive evaluation and for acknowledging both the novelty of our work and its contribution to advancing the field.

There are a few questions I would like addressed before I can recommend this paper for publication unconditionally. None are deal-breakers, but I think could potentially improve the manuscript if they are addressed and would provide additional valuable information to a reader in the field.
- Can the authors elaborate on what they expect the temporal structure of the XUV to be under their experimental conditions? In the manuscript they describe the high harmonic source being able to produce photon energies between 20-40 eV and can support isolated pulses of duration <= 180 as. However, given they are filtering only relatively low energy harmonics and are (I presume) far from the continuum cut-off region normally used for IAP generation (from the spectrum it looks like the authors have 2 near-discrete harmonics) then I would assume you would have some kind of pulse train, or at least some satellites? The reviewer was able to find a Master's Thesis by M. Trevisan which appears to describe the same/similar setup, where the transform limited duration was calculated to be 450 as, albeit with satellites about 15% of the main pulse height. I think including a brief explanation of this (or at least an upper limit for the XUV pulse duration similar to those stated for the UV pulses) in either the text or supplementary material could be useful in establishing what the current limits are for their temporal resolution (and potential for improvement in future!).
- Related to this: could the authors comment briefly on the reasons for the asymmetric shape/slope of the 2D sideband cross correlation trace?

We answer these two questions together, since they are fundamentally linked and are best understood in relation to one another.
In general, the tilt of a sideband spectrogram is determined by the chirp of both pulses, and a simultaneous measurement of SB+ and SB- is required to disentangle their respective contributions (B. Moio, et al., J. Phys. B: At. Mol. Opt. Phys. **54**, 154003 (2021)). In the present case, however, there are strong reasons to conclude that the observed tilt arises predominantly from the UV pulse.
First, an independent characterization of the UV generation sets its duration to Δt_{UV}= 3.1fs. If one applies the standard expression for the cross-correlation of Gaussian pulses,

$$\Delta t_{SB+} = \sqrt{\Delta t_{UV}^2 + \Delta t_{XUV}^2}$$

the experimentally observed sideband duration, $\Delta t_{SB+} = 3.2$ fs, is reproduced by assuming an **effective XUV pulse duration of 800 as. This value is fully consistent with the transform-limited duration expected for the portion of the XUV spectrum that contributes to the upper part of the SB+ signal.**

Second, the transform-limited duration of the UV pulse is $\Delta t_{UV} = 2.1$ fs. If one were to attribute the observed cross-correlation width of 3.2 fs to an unchirped UV pulse, the same formula would imply an effective XUV pulse duration of $\Delta t_{XUV} = 2.41$ fs, corresponding to a GDD of the order of 1 fs^2. In our experimental configuration, however, no plausible physical mechanism is expected to induce such a large chirp in the selected portion of the XUV spectrum. The attochirp is typically on the order of 10^{-2} fs^2, while femtochirp becomes relevant only when high harmonics are generated with a long IR driving pulse, which is not the case in our setup (M. Isinger, et al., Philos. Trans. R. Soc., A **377**, 20170475 (2019)).

These considerations justify our choice to perform the calculations by assuming transform-limited attosecond XUV pulses. Including the residual attochirp of the XUV radiation and the atomic delay introduced by the argon target would produce only a minor correction to the estimated IRF and would not alter the conclusions of the analysis.

In response to the Reviewer' requests for further clarification, we have added a new section to the Supplementary Information (Sect. 2 Instrumental response function) describing in greater detail the procedures used to determine the temporal characteristics of the pulses employed in the time-resolved experiments and the overall instrumental response function of the new beamline.

- If I am not mistaken, the ETF15 the authors utilize for electron spectroscopy can also be used in a positive ion mode. Given the challenge with signal/noise in the sideband measurements, I wonder if the authors considered/tried using ion yield measurements as an alternative/independent cross correlation scheme? I believe their total XUV+UV energies might be sufficient to conditionally ionise helium only in a XUV+UV two photon scheme, for example, and the mass selectivity could make the measurements less sensitive to background compared to electron detection.

We thank the Reviewer for this suggestion. While the ETF15 can indeed be used in ion mode, and the proposed XUV+UV ionization scheme in helium could in principle be implemented, we believe that this approach would provide a less informative observable. Since the Sn-filtered XUV spectrum excites helium without directly ionizing it, the resulting signal would be step-like, and the IRF could only be extracted from the steepness of its rising edge. By contrast, the sideband experiment presented here yields a far richer dataset: the two-dimensional spectrogram allows for a more detailed and robust comparison with numerical simulations, as shown below.

Other than these two questions I have only a few minor technical Points and suggestions for improvement:

- I think "Ultraviolet photoinduced dynamics" in the introductory sentence would read better as "Ultraviolet-photon induced electron dynamics" or "Electron dynamics induced by photoabsorption in the ultraviolet".

We've rephrased the sentence as "Ultraviolet-photon induced electron dynamics".

- In figure 1, FM (focusing mirror) and hollow core fibre (HCF) are not defined in the figure caption. While this is explained in text I think its always better if the captions are independently intelligible.

We've included the definitions in the caption.

- In figure 2 (a), I think it would be useful to indicate the expected electron kinetic energies of the MB and SB peaks in some way, or at least make the distinction between bound states and continuum electrons in some way (maybe by shading the continnum or bound states.

We've modified the figure, clarifying the distinction between bound and continuum states by shading.

- Figures 2 and 3 look a little congested as currently laid out. While I understand space is sometimes at a premium, I think a little more white space between some of the elements would go a long way to make the different elements more distinct from each other and improve the readability of the figures.

We've modified the figures, adding more white space between the elements.

- In the lower part of Fig. S1 it seems like the colour of data points in the pulse energy graphs don't have a 1:1 correspondence to the colours in the scales in the spectral plots above them. I think these subplots would benefit from either having independent colour scales or utilising some other way to indicate the pressure for each point. As it stands, it's non-trivial to gain anything too quantitative from the plot colours.

We thank the Reviewer for highlighting this. We've modified the figure clarifying the correspondence between plotted spectra and reported pulse energies.

Conclusion:
In summary, if the above are satisfactorily addressed through minor additions/changes, I would recommend that the editors approve the publication of this article.

We thank the Reviewer for the positive evaluation of our work. In the revised version of the manuscript, we have carefully considered and implemented all the Reviewer's suggestions.

Reviewer #2 (Remarks to the Author)

…. The results reported by Pini et al. in the present manuscript are therefore highly significant for ultrafast dynamics as it demonstrates time-resolved photoelectron spectroscopy with RDW UV and isolated attosecond XUV pulses, with an instrument response function of the order of just a few fs. The quality of the data is very good and the paper is well written. I think the manuscript can deserve publication in Nature Communications, after the questions raised by the use of ultrashort and therefore spectrally broad spectra for both the pump and the ionizing pulses are addressed in more detail.

We thank the Reviewer for the positive evaluation of our work, for recognizing the quality of the data, and for appreciating the overall presentation of the manuscript. We have carefully and seriously considered all the Reviewer's questions and comments regarding the experimental methodology adopted in this study, especially with respect to the use of ultrashort and consequently spectrally broad pump and ionizing pulses. These issues have been addressed in detail in the revised manuscript and in the point-by-point responses provided below.

Indeed, the few-fs UV pump pulse used for excitation shown in Fig. 2b spans 260-305 nm fwhm, which is over 700 meV fwhm bandwidth. As written by the authors in the manuscript, such a pump pulse can coherently excite multiple electronic (and a fortiori vibrational) states in a system, and I think that this point (very important for the future use of RDW UV pump in ultrafast spectroscopy and even "attochemistry"!) is not sufficiently discussed in the manuscript. Secondly, the probe pulse used for ionization displayed in Fig.2c spans over more than 4 eV. One can imagine that the ionization of a molecule with such a pulse leads to large, overlapping photoelectron bands. In the time-resolved photoelectron spectra presented in Fig. 3e, the authors seem to be able to identify features that are spectrally narrow compared to the ionizing probe pulse. To address these points, I would like the authors to:

We thank the Reviewer for this insightful comment. We agree that the possibility of coherently exciting multiple electronic states with such a broadband UV pump is an important point that deserves a more explicit discussion in the manuscript. To address this, we have expanded the relevant paragraph in the Introduction as follows:
"Moreover, the broad spectral bandwidth of the RDW UV pump does not only improve temporal resolution, but also enables the coherent excitation of multiple nearby electronic states and of the associated vibronic manifold. This is a crucial aspect for future ultrafast spectroscopy applications, because it allows access to non-stationary quantum states in which electronic and nuclear motion are coherently coupled from the earliest stages of the dynamics. In this respect, such pulses are highly relevant for emerging attochemistry-oriented strategies, where the preparation and probing of coherent electronic superpositions with large bandwidth may provide a route to influence photochemical evolution by acting directly on the initial electronic motion, before substantial decoherence or nuclear rearrangement takes place."

1) Justify the use of the same trajectories as in Ref. 38 in the simulation. Ref. 38 used a pump pulse with just 250 meV bandwidth fwhm compared to over 700 meV here. The trajectories from Ref. 38 were selected based on the spectrum of the excitation pulse of Ref. 38 which is different from the much broader spectrum used here. Can the broad pump spectrum coherently excite vibronic dynamics in AcAc?

The use of the same pool of trajectories as in Ref. 36 (Ref. 38 in the previous version of the manuscript) is justified by the absence of additional bright electronic states (other than S_2)

within the 260–305 nm spectral window. A sentence was added on pages 7-8 clarifying this point (a reference with AcAc's static UV absorption spectrum was also added, Ref. 40). However, the Reviewer correctly points out that the broader pump bandwidth could, in principle, access a wider range of vibrational excitations.

To address this point, we have analysed the pool of initial geometries sampled from Wigner distribution, and we found out that extending the pump window from 260–280 nm to 260–305 nm increases the number of accessible initial conditions by approximately 20%, while further extension up to 350 nm does not introduce additional contributions. This indicates that the original excitation window already covered the majority of the relevant vibronic phase space. To account for the broader pump spectrum, we have performed ~20 additional dynamics including initial excitations in the 280–305 nm region, which was not covered in Ref. 36, and incorporated these trajectories into the spectroscopic simulations.

The resulting time-resolved photoelectron spectra (tr-PES), shown in Fig. 3 of the main text, do not exhibit significant differences compared to those reported in the original manuscript, indicating that the broader pump bandwidth has a negligible impact on the underlying dynamics and supporting the validity of using the original trajectory pool. These new simulations are now discussed and compared to the original pool from Ref. 36 in the Supplementary Information (Sec. 2.1)

2) Along the same lines, how are the timescales and branching ratios discussed on p.10 and shown in Fig.4 dependent on the initial excited state population (and therefore on the spectral bandwidth of the pump pulse)? The authors write multiple times in the manuscript that their experiment in AcAc enables the observation of dynamics that have remained "unresolved" or "invisible" due to a lack of temporal resolution… But is it really the same dynamics if a narrow or broad pump pulse is used to trigger them?

To address this point, we have compared the population dynamics of the original trajectory set from Ref. 36 with the extended set including additional excitations in the 280–305 nm region. The results show negligible differences between the two datasets, as shown in Fig. R1 (we added this figure also in the Supplementary Information, Fig. S7).

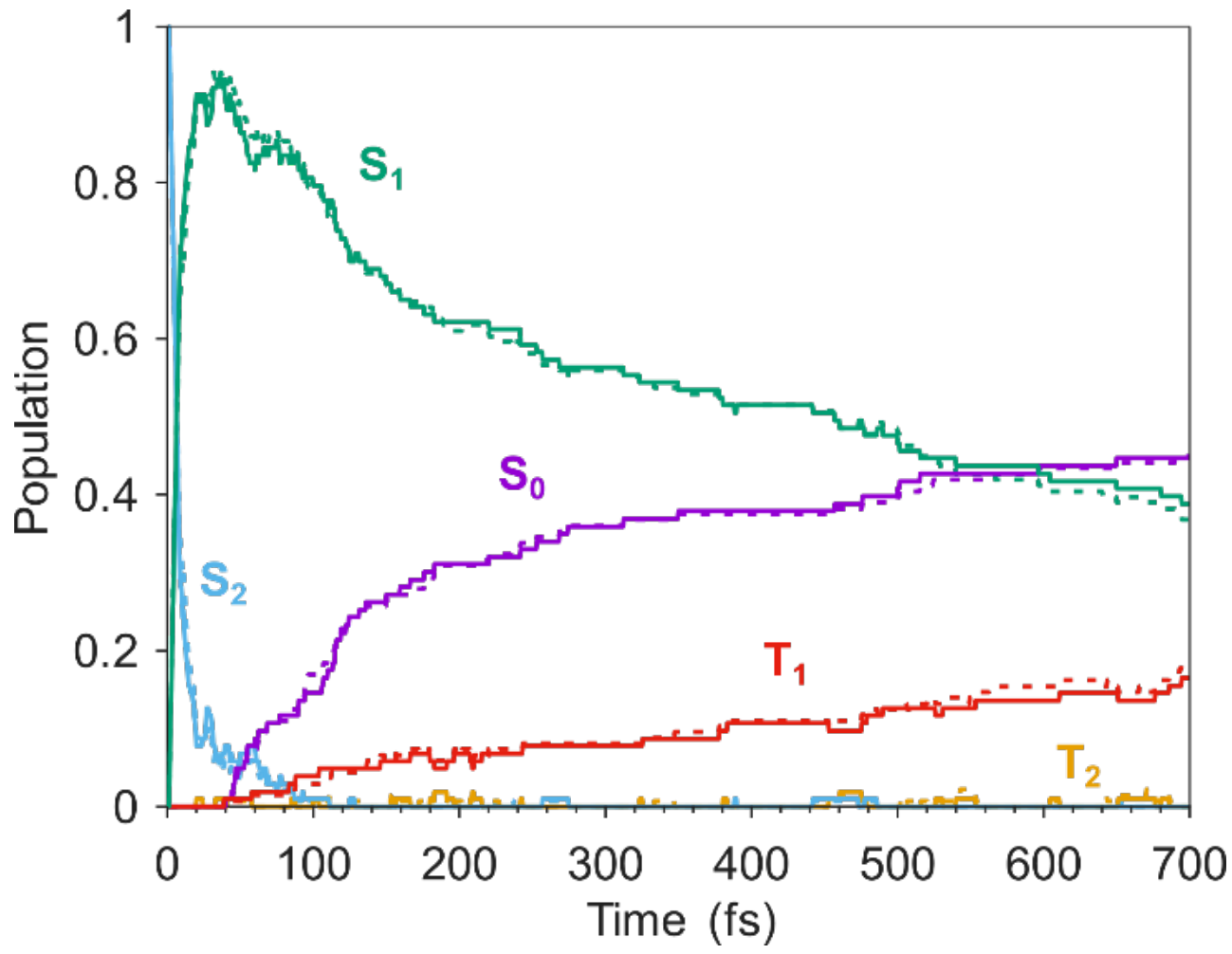

Figure R1: Comparison of the population dynamics using the 103 trajectory-ensemble from reference [6] (full lines) and the 136 trajectory-ensemble that accounts for the broader excitation pulse of the present experiment (dashed lines). S_2 and S_1 denote the singlet excited states discussed in the main text, T_2 and T_1 the triplet states, and S_0 the ground state.

3) The authors should show and comment the static XUV only photoelectron spectrum of the molecule. From the data in Fig. 3e, it looks like the part of the XUV spectrum peaked around

22 eV is mainly contributing to the ionization. However, a discussion on the ability to get "good" spectral resolution in the photoelectron spectra despite using a 4-eV broad ionizing pulse is needed.

We show and comment below the static XUV spectrum of the molecule. A corresponding section (Sect. 5) has been added to the Supplementary Information.

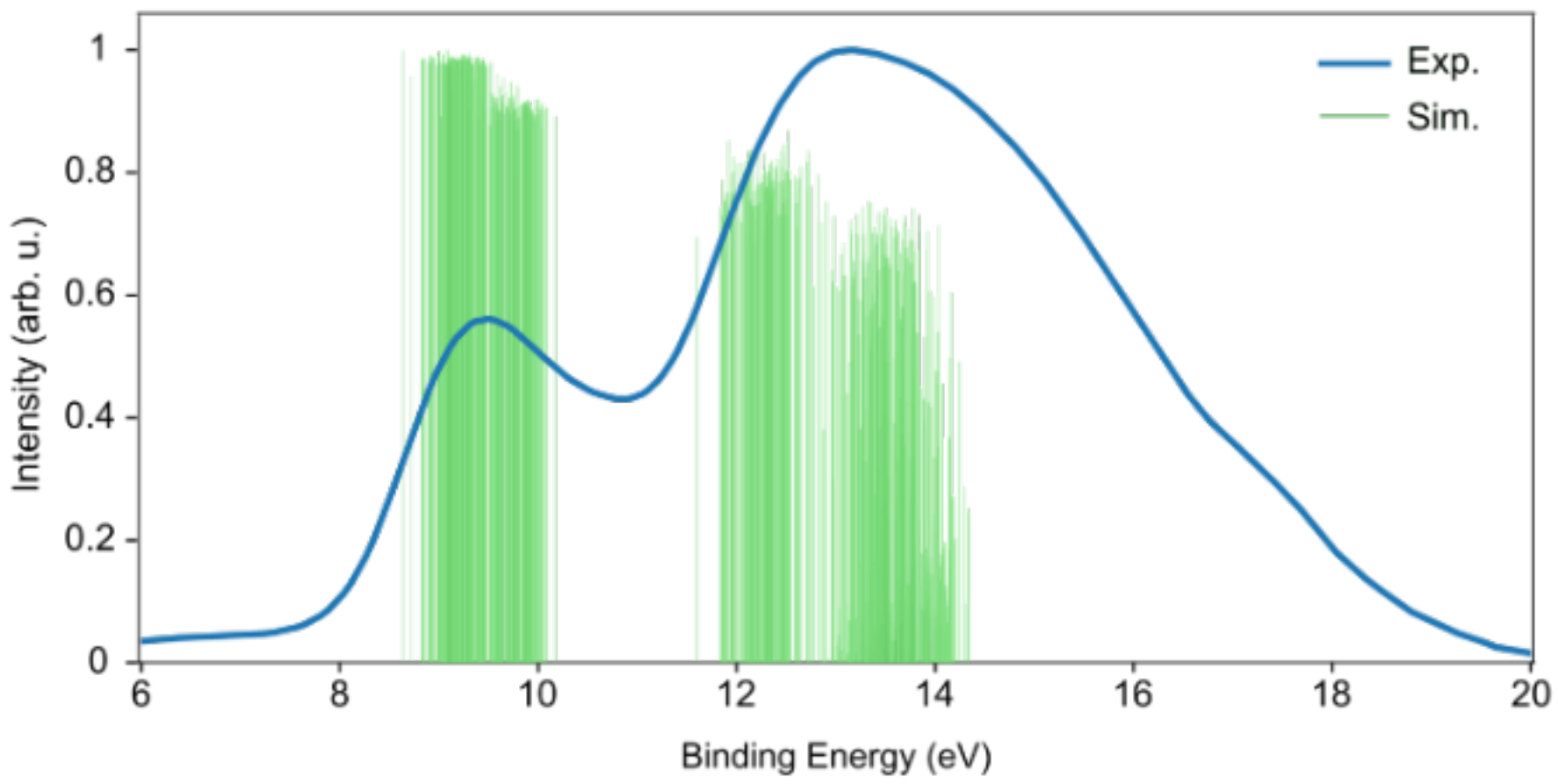

Figure R2: Static XUV spectrum of acetylacetone. The experimental spectrum is shown in blue, while the green sticks represent the simulated spectral contributions.

The calculated static PES displays four main ionization channels from the ground state (S0), located at 8.92 eV (S0->D0), 9.47 eV (S0->D1), 12.19 eV (S0->D2), and 13.25 eV (S0->D3). The electronic structure analysis shows that each ionic state is dominated by a single leading configuration (corresponding to ionizations from π HOMO, n HOMO-1, π HOMO-2 and π HOMO-3 orbitals, respectively), indicating a largely single-hole character with moderate configuration mixing. In view of the relatively broad bandwidth of the XUV radiation exploited in the experiment, the experimental static spectrum displays only two main features, corresponding to the first pair (S0->D0 and S0->D1) and the second pair (S0->D2 and S0->D3) of ionization channels, respectively.

4) Along the same lines, in the dressed photoelectron spectrum used for the characterization of the instrument response function shown in Fig. 2d, the width of SB+ shown is much smaller than the width of the XUV pulse. Because of the large XUV spectrum, some of SB+ probably overlaps with the MB that cannot be measured for technical reasons. Therefore, contrary to Refs. 32 and 33 which used more "monochromatic" XUV, it seems that not all of SB+ signal is integrated to determine the IRF. The IRF is thus probably longer than the 3.2 fs given here?

The Reviewer is correct: not the entire SB+ signal is integrated to determine the IRF. In fact, part of the SB signal overlaps with the direct XUV ionization signal and cannot be measured simultaneously, owing to the excessively large difference in signal dynamics. **As we show below, this leads to an overestimation of the IRF**.
We performed Strong-Field Approximation (SFA) calculations (L. B. Madsen, "*Strong-field approximation in laser-assisted dynamics*", Am. J. Phys. **73**, 57 (2005)) using as input the experimental UV and XUV spectra reported in Fig. 2(b) and 2(c), respectively, of the main manuscript. In the simulations, no spectral phase was assigned to the XUV pulse. As discussed below, this approximation does not represent a significant limitation for the present analysis. To reproduce the experimental conditions under which h the SB+ spectrogram shown in Fig. 2(d) was measured, the UV peak intensity was set to $\sim 7\times10^{11}$ W/cm^2. In addition, a parabolic

spectral phase was applied to the UV pulse, corresponding to a group-delay dispersion (GDD) of 1.5 fs^2. Under these conditions, the UV pulse reaches a FWHM duration of 3.08 fs, in very good agreement with the pulse duration expected from our previous characterization (M. Reduzzi et al., Opt. Express **31**, 26854 (2023)).

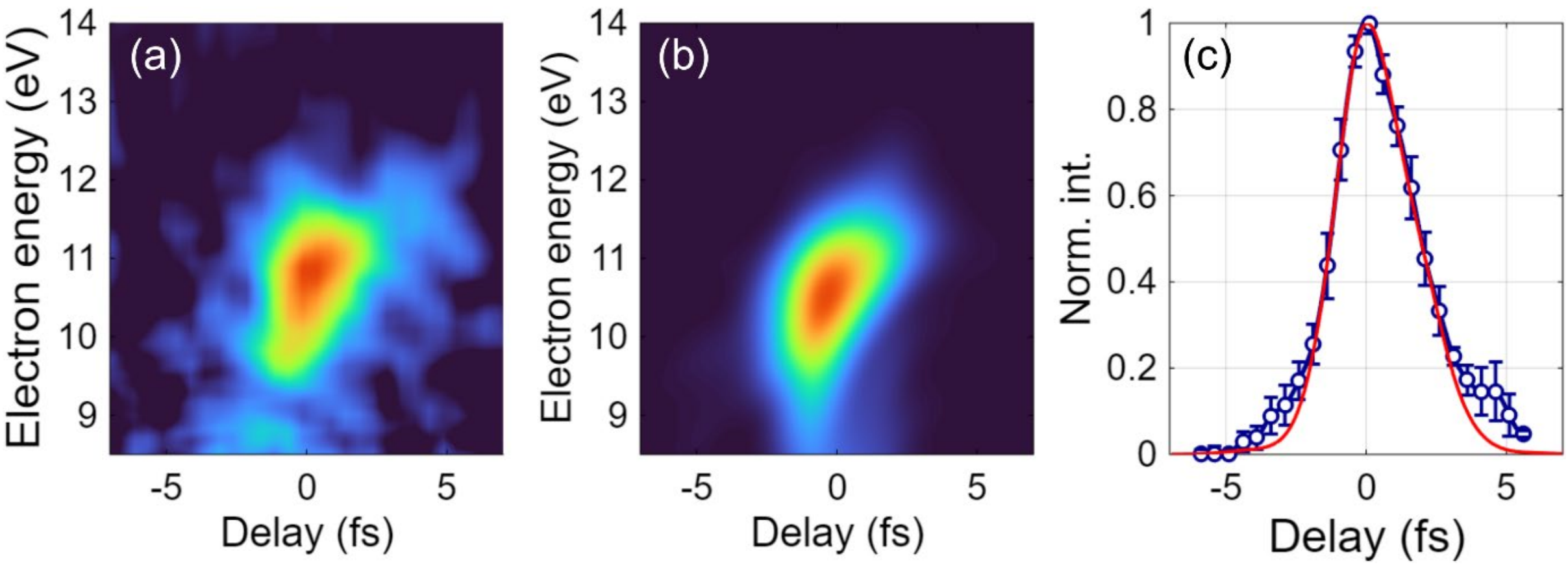

Figure R3: (a) Experimental spectrum, identical to that shown in Fig. 2(d) of the main text. (b) Calculated spectrum. (c) Comparison of the delay-dependent SB+ signal extracted from experiment and simulation: blue open circles with error bars show the experimental data, identical to those in Fig. 2(e) of the main text, while the red solid curve shows the simulated profile, shifted by 500 as toward positive delay for clarity.

The experimental and calculated delay-dependent photoelectron spectrum of the sideband SB+ signal are shown in Figs. R3(a) and R3(b), respectively; the experimental data are the same as those presented in Fig. 2(d) of the main text. In Fig. R3(c), the blue open circles with error bars represent the experimental data, identical to those shown in Fig. 2(e) of the main manuscript, whereas the red solid curve corresponds to the simulated profile (shifted by 500 as toward positive delay for clarity). Overall, the agreement between experiment and simulation is very satisfactory in all panels, and notably this is achieved without introducing any additional adjustable parameters beyond those independently determined from the experimental conditions.

To elucidate the physical origin of the signal observed in Fig. R3(a), we decomposed the calculated spectrogram according to the procedure introduced in M. Lucchini, *Floquet Interpretation of Dynamic Interference in Laser-Assisted Photoemission*, Phys. Rev. A **112**, 043120 (2025). The results are reported in Fig. R4. Panel (a) shows the contribution associated with the process involving absorption of one XUV photon and emission of one UV photon, i.e. the SB- channel. Panels (b) and (c) display the spectrograms associated with direct XUV ionization (main band, MB) and with the first upper sideband (SB+), respectively. In Fig. R4(d), we report the corresponding delay-integrated spectra obtained from Fig. R4(b) (solid blue curve) and Fig. R4(c) (solid red curve), together with the experimental profile extracted from Fig. R3(a) (solid orange curve with shaded area). This comparison shows that the SB+ signal spectrally overlaps with the MB contribution. In the experiment, however, this overlapping region cannot be accessed because the detector saturates. To facilitate the comparison, the MB profile has been shifted upward by one UV-photon energy, yielding the dashed blue curve in Fig. R4(d). Its qualitative similarity to the SB+ profile (solid red curve) indicates that the spectral region of SB+ used to estimate the instrumental response function (IRF), namely the interval delimited by the horizontal black dashed lines, originates predominantly from the absorption of one XUV photon associated with the peak labeled A in Fig. R4(d), corresponding to approximately 22 eV in the original XUV spectrum.

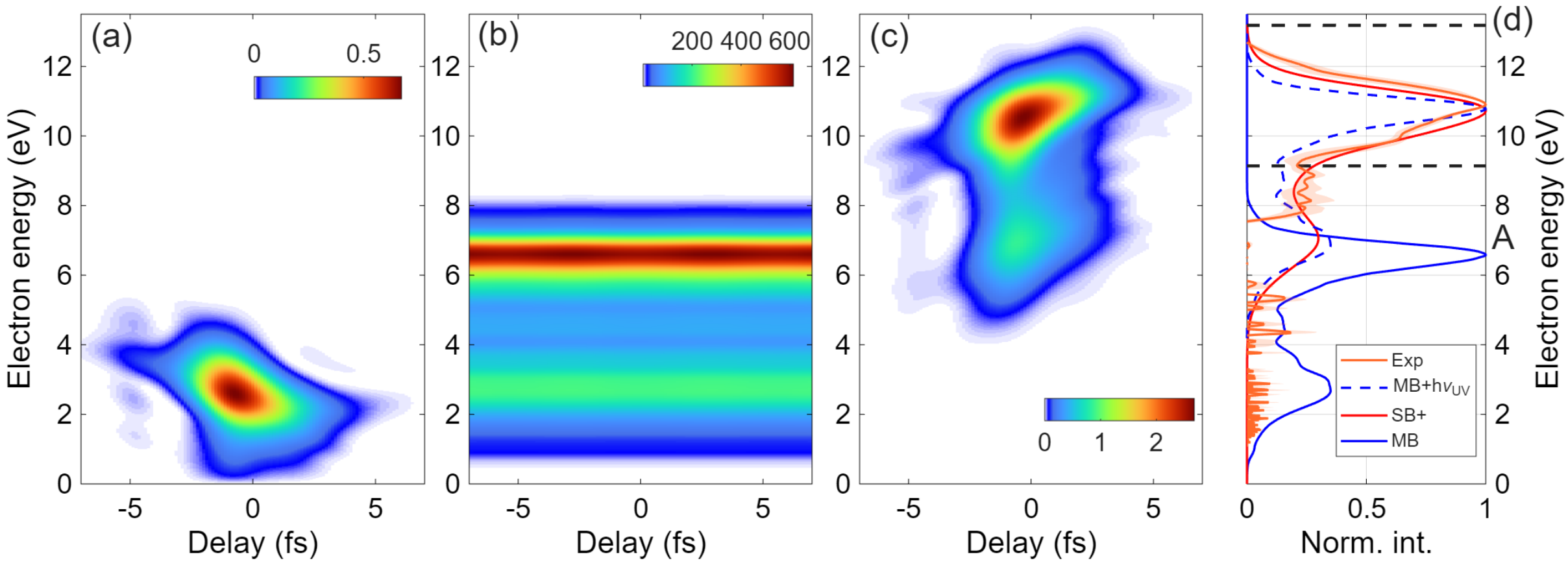

Figure R4: Floquet decomposition of the total SFA spectrogram. (a) Signal produced by absorption of one XUV photon and emission of an UV photon (SB-). (b) Signal associated with direct XUV ionization (MB). (c) Signal produced by absorption of one XUV photon and one additional UV photon (SB+). (d) Comparison between the delay-integrated spectrograms and experiment. The experimental SB+ integral is reported in orange with shaded area. The integral of the MB is indicated with solid blue curve. The SB+ integral is reported in solid red, while the dashed blue curve is the MB profile shifted in energy by one UV photon. The black dashed lines mark the energy region with no overlap (back-ground free), used in the paper to estimate the signal cross-correlation.

At sufficiently low UV intensity, the delay-integrated SB+ spectrogram corresponds to the convolution of the spectral intensities of the XUV and UV pulses, whereas the energy-integrated SB+ trace corresponds to the convolution of their temporal intensities (B. Moio, et al., J. Phys. B: At. Mol. Opt. Phys. **54**, 154003 (2021)). In the present analysis, the energy integration used to retrieve the IRF is performed only over the upper portion of the SB+ signal, i.e. over the region associated with the dominant peak of the XUV spectrum (see Fig. R5(a)).

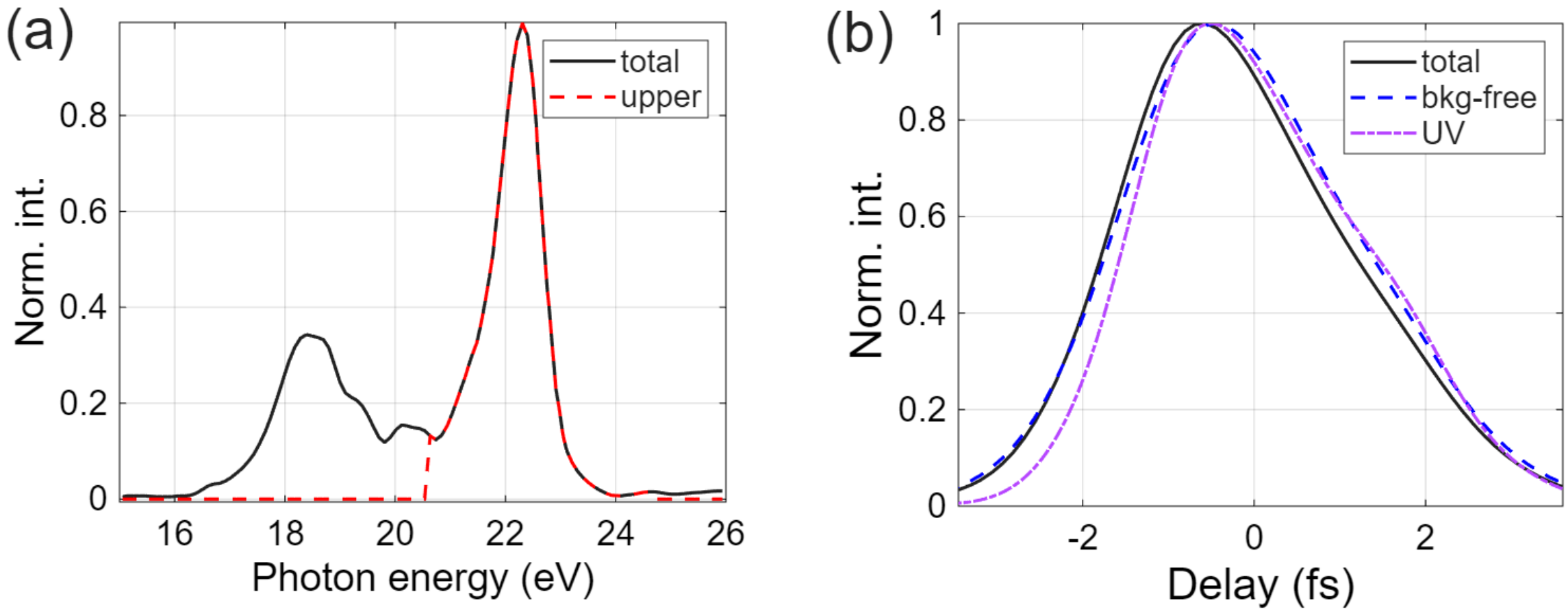

Figure R5: (a) Experimental XUV spectrum, identical to that shown in Fig. 2(c) of the main text (solid black curve), together with the portion of the XUV spectrum that gives rise to the measurable part of the SB signal (dashed red curve). (b) Corresponding normalized temporal profiles obtained by integrating the SB over the full spectral region (solid black) and over the background-free spectral region that is experimentally accessible (dashed blue curve), shown together with the UV pulse profile (dash-dotted purple curve

Therefore, only a reduced portion of the XUV spectrum contributes to the measured signal. Since this portion is spectrally narrower than the full XUV pulse, the retrieved cross-correlation width should be regarded as an upper limit to the actual one (solid black and dashed blue curves in Fig. R5(b)). This effect is further illustrated by the transform-limited pulse durations associated with the relevant spectral windows. When the full XUV spectrum is considered, the transform-limited duration is approximately 500 as (solid black curve in Fig. R5(a)), whereas restricting the analysis to the upper spectral portion increases the transform-limited duration to about 1 fs (dashed red curve in Fig. R5(a)). Despite this reduction in bandwidth, the resulting broadening of the energy-integrated SB+ trace, and thus of the retrieved IRF, is only about 200

as. Therefore, the temporal resolution inferred from the experimentally accessible part of the SB+ signal remains a reliable estimate of the overall instrument response. It's worth noting that, regardless of the portion of XUV spectrum considered, the IRF is largely dominated by the UV pulse profile (dash-dotted purple curve in Fig. R5(c)).

In addition, the SB+ appears tilted in Fig. 2d – is this the sign of a chirp in either the XUV or UV pulses (or both)?

In general, the tilt of a sideband spectrogram is determined by the chirp of both pulses, and a simultaneous measurement of SB+ and SB- is required to disentangle their respective contributions (B. Moio, et al., J. Phys. B: At. Mol. Opt. Phys. **54**, 154003 (2021)). In the present case, however, there are strong reasons to conclude that the observed tilt arises predominantly from the UV pulse.
First, an independent characterization of the UV generation sets its duration to Δt_{UV}= 3.1fs. If one applies the standard expression for the cross-correlation of Gaussian pulses,

$$\Delta t_{SB+} = \sqrt{\Delta t_{UV}^2 + \Delta t_{XUV}^2}$$

the experimentally observed sideband duration, Δt_{SB+} = 3.2fs, is reproduced by assuming an effective XUV pulse duration of 800 as. This value is fully consistent with the transform-limited duration expected for the portion of the XUV spectrum that contributes to the upper part of the SB+ signal.
Second, the transform-limited duration of the UV pulse is Δt_{UV}= 2.1 fs. If one were to attribute the observed cross-correlation width of 3.2 fs to an unchirped UV pulse, the same formula would imply an effective XUV pulse duration of Δt_{XUV}= 2.41 fs, corresponding to a GDD of the order of 1 fs^2. In our experimental configuration, however, no plausible physical mechanism is expected to induce such a large chirp in the selected portion of the XUV spectrum. The attochirp is typically on the order of 10^{-2} fs^2, while femtochirp becomes relevant only when high harmonics are generated with a long IR driving pulse, which is not the case in our setup (M. Isinger, et al., Philos. Trans. R. Soc., A **377**, 20170475 (2019)).
These considerations justify our choice to perform the calculations by assuming transform-limited attosecond XUV pulses. Including the residual attochirp of the XUV radiation and the atomic delay introduced by the argon target would produce only a minor correction to the estimated IRF and would not alter the conclusions of the analysis.

In response to the Reviewer' requests for further clarification, we have added a new section to the Supplementary Information describing in greater detail the procedures used to determine the temporal characteristics of the pulses employed in the time-resolved experiments and the overall instrumental response function of the new beamline.

5) The n-pi* and pi-pi* states should be distinguishable in the photoelectron spectrum because they would primarily ionize to different states of the cation (see e.g. DOI 10.1021/jacs.4c02886). This is suggested by the decompositions of the simulated tr-PES in Fig.S4 b and c, but a more explicit discussion of this point would be valuable in the context of tr-PES with XUV pulses where multiple cationic states are accessible.

We thank the reviewer for this comment. Although we were referring to the decomposition, Fig. S4 (now Fig. S8) was not explicitly mentioned in the main text. We followed the Reviewer's suggestion and addressed this point at several places in the section "UV pump – XUV probe in Acetylacetone: …" of the main manuscript, including a reference to Fig. S8.

In addition, I have a few minor comments:

6) What do you mean by “full and aborted H transfer” on p. 10? Do you refer to the “33%” path in Fig. 4 as full H transfer and the other paths in Fig. 4 as aborted H transfers?

Yes. To clarify this point, we added a sentence in the main manuscript: “As discussed in the Supplementary Information, complete O-H … O → O … H-O transfer is obtained in 33% of the cases.”

7) The RDW spectra in Fig. 1b seem to correspond to the Argon spectra in Fig. S1, but the legend of Fig.1 in the main text reads “neon” and so does the text on p.5. Please correct the gas used.

The correct gas used is argon. The caption of Fig. 1 and the text on page 5 have been corrected.

8) The methods section only gives the maximum estimated UV peak intensity. For reproducibility purposes, please add the estimated peak intensities in the experiments reported in Fig. 2d and 3e.

The estimated peak intensity of the experiments has been reported at page 6, and at page 7 it is clarified that the pulse used for the AcAc experiment is the same used for the sidebands, also in terms of energy and intensity.

9) When discussing the molecular structure of AcAc (shown in Fig. 3a), a precision that the enolic form is dominant in the gas phase would benefit a non-specialist community.

A sentence was added at the beginning of the AcAc section on page 7, providing more context on AcAc’s keto-enol tautomerism.

10) The colors used in Fig. 4 are hardly distinguishable if the figure is printed in shades of grey.

We agree with the Reviewer. Unfortunately, despite trying different colour palettes, we could not find any that resulted in a markedly improved readability in shades of grey. For this reason, we decided to keep the original one.

Reviewer #3 (Remarks to the Author)

… Overall, the manuscript is well written, the technical execution impressive, and the demonstrated timing capability will be broadly useful for ultrafast laboratories pursuing studies of sub–10 fs chemical dynamics. That said, there are several important questions regarding the acetylacetone study that need to be clarified in order for the manuscript to be suitable for publication in Nature Communications.

We thank the Reviewer for the positive evaluation of our work, for acknowledging the technical quality of the manuscript, and for highlighting the potential broad impact of the demonstrated timing capability for ultrafast laboratories studying sub-10 fs chemical dynamics. We have carefully and seriously considered all of the Reviewer's questions and comments concerning the acetylacetone study, and we have revised the manuscript accordingly. Below, we provide a detailed response to each point raised.

Major questions

1. Broadband excitation vs. single-state ("prepared S_2") narrative: The manuscript emphasizes the broad bandwidth of both the UV pump and XUV probe pulses and frames this capability as enabling precise excitation/probing of coherent superpositions central to "attochemistry." However, the mechanistic interpretation for acetylacetone appears to be closely aligned with earlier work—primarily a refinement in temporal resolution rather than a qualitatively new dynamical picture. Given that the bandwidth of the UV pulse spans multiple eV, the authors should (i) justify why the initially prepared wave packet is dominated by a single electronic state (S_2) rather than a mixture of different excited states accessible within the photon energy range covered by the excitation pulse, and (ii) discuss why signatures of electronic coherence are not apparent in the observables.

(i) The initially prepared wave packet is dominated by a single electronic state (S_2) rather than a mixture of different excited states is justified by the absence of additional bright electronic states (other than S_2) within the 260–305 nm spectral window. A sentence was added on page 7 clarifying this point (a reference with AcAc's static UV absorption spectrum was also added, Ref. 40). However, the Reviewer correctly points out that the broader pump bandwidth could, in principle, access a wider range of vibrational excitations.

To address this point, we have analysed the pool of initial geometries sampled from Wigner distribution, and we found out that extending the pump window from 260–280 nm to 260–305 nm increases the number of accessible initial conditions by approximately 20% (while further extension up to 350 nm does not introduce additional contributions). This indicates that the original excitation window already covered the majority of the relevant vibronic phase space. To account for the broader pump spectrum, we have performed ~20 additional dynamics including initial excitations in the 280–305 nm region, which was not covered in Ref. 36, and incorporated these trajectories into the spectroscopic simulations.

The resulting time-resolved photoelectron spectra (tr-PES), shown in Fig. 3 of the main text, do not exhibit significant differences compared to those reported in the original manuscript, indicating that the broader pump bandwidth has a negligible impact on the underlying dynamics and supporting the validity of using the original trajectory pool. These new simulations are now discussed and compared to the original pool from Ref. 36 in the Supplementary Information (Sec. 2.1)

(ii) The absence of electronic coherences is a consequence of the absence of additional bright electronic states (other than S_2) within the 260–305 nm spectral window. A sentence was added

on page 7 clarifying this point (a reference with AcAc's static UV absorption spectrum was also added, Ref. 40).

2. Role of triplet states and early-time ISC: The mechanistic model and simulations appear to neglect triplet pathways. While singlet -> triplet intersystem crossing (ISC) is often reported on ~1.5 ps timescales, the earlier work used for comparison (Severino et al., J. Am. Chem. Soc. 2025, 147, 30785–30793) suggests some population transfer to T_1 within the first ~100 fs. Can the authors clarify the rationale for excluding the triplet states here and state explicitly whether this experiment is sensitive to early ISC (e.g., via distinct photoelectron signatures), whether any such contribution is unresolved within the fit, or whether it is spectroscopically overlapped with singlet features?

As the Reviewer correctly points out, intersystem crossing (ISC) in photoexcited AcAc can occur on sub-picosecond timescales. Indeed, triplet states are explicitly included in our dynamical simulations (see Ref. 36 (S. Severino *et al.*, Ref. 38 of the previous version of the manuscript), where ISC is observed to begin within a few hundred femtoseconds. In the present work, however, we have restricted the spectroscopy simulation to the first 100 fs time window, when the population of the triplet manifold remains very small, and its contribution to the total Tr-PES signal is therefore not detectable. Furthermore, as discussed in Ref. 36, there is an almost perfect energetical overlap of the S1 and T1 signals in the region of the potential energy surface where ISC. As a result, even if a minor triplet population were present at early times, it would not produce a distinct or separable signature in the measured Tr-PES.
On this basis, we conclude that while early-time ISC is included in the simulations, it does not play a measurable role within the temporal and spectral window considered in this study.

3. Experiment–theory mismatches: Experiment and simulations show very good agreement in terms of the timescales of the extracted components (Fig. 3(g)). However, the readers would benefit from a more explicit discussion of where the simulated trPES deviates from the experiment (e.g., binding-energy positions, relative intensities in Fig. 3(f,i)) and why this is the case.

The remaining discrepancies between simulated and experimental trPES signals, most notably in binding-energy positions and relative intensities (Fig. 3(f,i)), can be attributed to technical aspects of the simulation protocol.
The simulated spectral features are narrower than the experimental ones by design, in order to better resolve the vibronic structure underlying the photoelectron signal. At the same time, the first band in the simulations (S_2 signal) is slightly blue-shifted compared to the experiment. This originates from the procedure used to account for the finite pump bandwidth: the simulated signal is averaged (via convolution) over the first 3 fs of dynamics to mimic the associated spectral uncertainty. While this provides a practical approximation, it introduces an artificial blue-shift of the initial signal ($t = 0$) with respect to the Franck–Condon (FC) energy. Indeed, the FC vertical ionization energy itself (4.16eV) is in much better agreement with the experimental binding energy of the first peak. We also note that this approximate treatment of the finite pulse duration likely contributes to the overestimation of the intensity of the first signal relative to the experiment.

We have added a sentence at page 9 of the main text and a section of the Supplementary Information to clarify this point.

Minor comments

1. The simulated spectra are convolved with a temporal FWHM of 3 fs and an energy FWHM of 0.59 eV. Please justify the 0.59 eV broadening chosen for the effective experimental energy resolution.

The value of 0.59 eV arises from a technical convention used in our implementation. Specifically, the temporal broadening is defined in terms of full width at half maximum (FWHM), whereas the energy broadening is specified as a standard deviation (σ). In the simulations, we employ a temporal broadening of 3 fs (FWHM) and an energy broadening of 0.25 eV (σ), which corresponds to an effective FWHM of 0.59 eV. The choice of these phenomenological parameters does not reflect a fundamental limitation, but rather a practical choice to reproduce the experimental conditions.

2. The manuscript argues that many trPES experiments are limited to ~10 fs resolution, impeding access to sub–10 fs dynamics. It would be informative to convolve the simulated trPES with 10 fs (in addition to 3 fs) to show which qualitative signatures survive and to clarify why acetylacetone is a particularly compelling system for showcasing the improvement.

Following the Reviewer's recommendation, we convoluted the simulated trPES traces with a Gaussian instrument response function (IRF) of 10 fs (FWHM), in addition to the 3-fs case discussed in the main text. The resulting traces are shown in Fig. R6. Already at a purely qualitative level, the 10 fs convolution markedly suppresses the visibility of both the oscillatory modulations and the earliest steps of the temporal evolution. This qualitative impression is fully supported by the quantitative analysis, which reveals substantial limitations in resolving the initial dynamics. Specifically, a global fit of the 3-fs convoluted trace yields an initial relaxation time constant of 8.1 fs, whereas the same analysis applied to the 10-fs convoluted trace returns a first component with a relaxation time constant of approximately 10 fs, i.e. effectively constrained by the IRF itself. This demonstrates that a temporal resolution of 10 fs is not sufficient to reliably resolve the earliest stage of the dynamics.

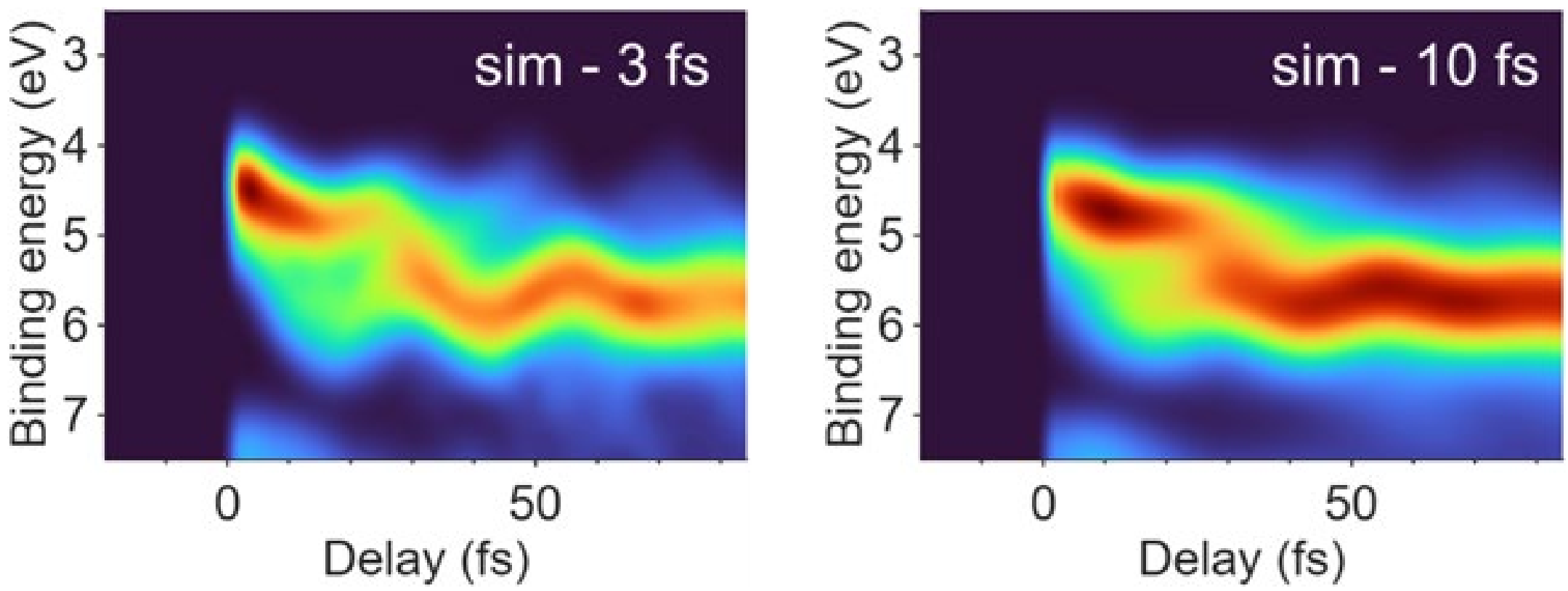

Figure R6: Simulated trPES traces convolved with a Gaussian instrument response function of 3-fs (left panel) and 10-fs (right panel) FWHM

To evaluate the impact of the broader IRF under experimental conditions, we applied the same 10-fs convolution to the experimental data. The results are reported in Fig. R7. In this case, the degradation is even more pronounced: even on a qualitative basis, the distinct features governing the early-time dynamics become difficult to disentangle. We carried out a global fit analysis of the experimental data convoluted with a 10 fs IRF. Unlike the 3 fs case discussed in the manuscript, where three components are required to properly capture the dynamics, the 10 fs convoluted data can be adequately described using only two components. This result is

fully consistent with the observations of Severino *et al*. [Ref. 36], whose measurements at 20 fs temporal resolution similarly revealed only two dynamical components.
Taken together, these results strongly support our original conclusion that sub-10 fs temporal resolution is essential in this system to disentangle the earliest dynamical processes, and that acetylacetone represents a particularly stringent and illustrative benchmark for this purpose.

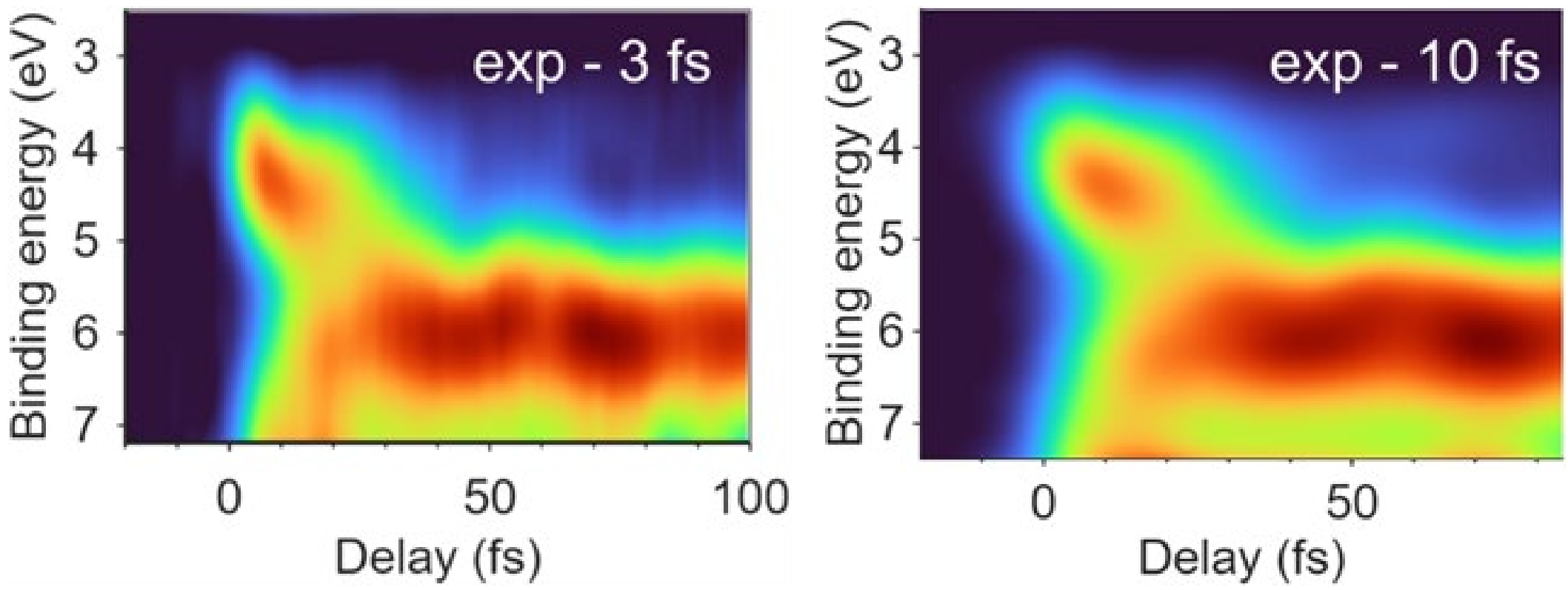

Figure R7: Measured trPES traces convolved with a Gaussian instrument response function of 3-fs (left panel) and 10-fs (right panel) FWHM

3. The authors state that they "directly" resolve two CI passages. Improved time resolution can certainly reveal features that would otherwise be blurred, but the manuscript should define what is meant by "direct" in this context.

We thank the Reviewer for this comment. As we feel the word "direct" is not necessary to convey the intended message, it's been removed both in the abstract and in the conclusions.

Reviewer #4

I co-reviewed this manuscript with one of the reviewers who provided the listed reports. This is part of the Nature Communications initiative to facilitate training in peer review and to provide appropriate recognition for Early Career Researchers who co-review manuscripts.

We thank the Reviewer for the work.
